# Supplementary material for: Reduction of H3K9 methylation by G9a inhibitors improves the development of mouse SCNT embryos
Source: Stem Cell Reports. 2024 May 9;19(6):906–21. doi: 10.1016/j.stemcr.2024.04.003 (PMC11390627; doi:10.1016/j.stemcr.2024.04.003)
Supplement: Document S1. Supplemental experimental procedures, Figures S1–S6, and Tables S2, S3, S5, and S6 [file mmc1.pdf]

Stem Cell Reports, Volume *19*

Supplemental Information

Reduction of H3K9 methylation by G9a inhibitors improves the development of mouse SCNT embryos

Shogo Matoba, Daiki Shikata, Fumiyuki Shirai, Takaki Tatebe, Michiko Hirose, Akiko Nakata, Naomi Watanabe, Ayumi Hasegawa, Akihiro Ito, Minoru Yoshida, and Atsuo Ogura

Supplemental experimental procedures

In vitro fertilization

B6N female and DBA/2 male mice at the age of 9–12 weeks were used to generate IVF control embryos in a BDF1 background. Briefly, spermatozoa were harvested from the epididymis of adult DBA/2 males and incubated in human tubal fluid (HTF) drops for 1 h at 37°C under 5% CO_2 in humidified air. Cumulus–oocyte complexes (COCs) were collected from the oviducts of B6N females that had been superovulated by injection of anti-inhibin serum (gift of Dr. Gen Watanabe at Tokyo University of Agriculture and Technology) followed by 7.5 IU of human chorionic gonadotropin (hCG; Aska Pharmaceutical Co. Ltd.) at 48 h intervals. The COCs were isolated 15–17 h after the hCG injection and incubated in HTF containing 0.04% glutathione (L-glutathione reduced, Sigma Aldrich #G6013) for 1 h before insemination. After preincubation, the activated spermatozoa were introduced into HTF drops containing COCs to initiate insemination. Five to six hours after the initiation of insemination, the fertilized zygotes were washed and transferred to drops of potassium-enriched simplex optimization medium (KSOM).

Donor cell preparation

Primary MEFs were derived from BDF1 male mouse embryos at 13.5 days post coitum. After removal of the head and all organs, minced tissue from the remaining corpus was dissociated in 500 ml of 0.25% trypsin with 1 mM EDTA (Thermo Fisher Scientific #25200056) for 10 min at 37°C. The cell suspension was washed twice with 10% fetal bovine serum (FBS) DMEM and plated in 100-mm dishes, and the plates were cultured at 37°C. Two days later, MEFs were harvested and frozen. Frozen stocks of MEFs were thawed and used for experiments after one passage.

Cumulus cells were collected from adult BDF1 female mice treated with superovulation by injection of 7.5 IU of pregnant mare serum gonadotropin (PMSG; Millipore #367222) and 7.5 IU of hCG (Millipore #230734). COCs were collected from the oviducts 15–17 h after the hCG injection and were treated briefly with HEPES-buffered KSOM containing 300 U/ml bovine testicular hyaluronidase (Calbiochem #385931) to obtain dissociated cumulus cells.

Sertoli cells were collected from the testes of 3–7-day-old BDF1 male mice as described previously (Matoba et al., 2011, 2018). Testicular masses were incubated in phosphate-buffered saline (PBS) containing 0.1 mg/ml collagenase (Thermo Fisher Scientific #17104-019) for 30 min at 37°C and then treated for 5 min with 0.25% trypsin with 1 mM EDTA at room temperature. The dissociated cells were washed four times with PBS containing 3 mg/ml bovine serum albumin (BSA) and then suspended in HEPES-buffered KSOM medium.

Embryo transfer

Two-cell stage SCNT embryos were transferred to the oviducts of pseudopregnant (embryonic day (E) 0.5) ICR females. The pups were recovered by caesarean section on the day of delivery (E19.5) and nursed by lactating ICR females.

Reverse Transcription and Real-Time PCR (related to Figure S1B and S1C)

Two-cell stage embryos were collected at 28 hpi or hpa. Five embryos were pooled as a single sample for each condition. cDNA was directly synthesized with a Cells-to-cDNA kit (Thermo Fisher Scientific, AM1722) with oligo-dT primer. RT-qPCR was then performed using PowerUp SYBR Green Master Mix (#A25742; Thermo Fisher Scientific) with the QuantStudio 7 system

(Thermo Fisher Scientific). The Ct values were normalized to that of external control RNA (Lambda polyA; Takara, #3789). The expression level of each gene was further normalized for IVF control as 1. The primers used for RT-qPCR are as follows; Zscan4d-RT-F1: TCTTTCTGGTTGGCAGCTTT, Zscan4d-RT-R1: GCCTCTGTCAGGACCACTGT, Zscan4a-RT-F1: CCTTTCATCCCACCAGAGAA, Zscan4a-RT-R1: AAGGCTTTTCTGGCATGTGT, Obox3-RT-F1: AGATGAGCTCTGGCAAAGGA, Obox3-RT-R1: TTGGACTGGTCAAGGACCTC, Obox6-RT-F1: TCCTGATTCCTTACCCGTTG, Obox6-RT-R1: CCTGATAATGAGGCGGAGAA, Obox8-RT-F1: CACCCCCAAGAACGTCTAAA, Obox8-RT-R1: GCTAGGGTTGGGTCACAGAA.

Histology (related to Figure S6F)
Placentae collected from E19.5 embryos derived from IVF or SCNT were fixed in 4% paraformaldehyde (PFA) at 4°C overnight and embedded in paraffin blocks. Sections (4 mm in thickness) were subjected to periodic acid Schiff (PAS) staining. Each stained section was scanned on a BZ-9000 microscope (Keyence Japan) and processed using BZ-II Analyzer software (Keyence Japan). At least three placentae for each condition were examined. Representative images of the placenta for each sample were combined into a single panel by adjusting the scale (Figure S6F).

Supplemental Figures

Figure S1

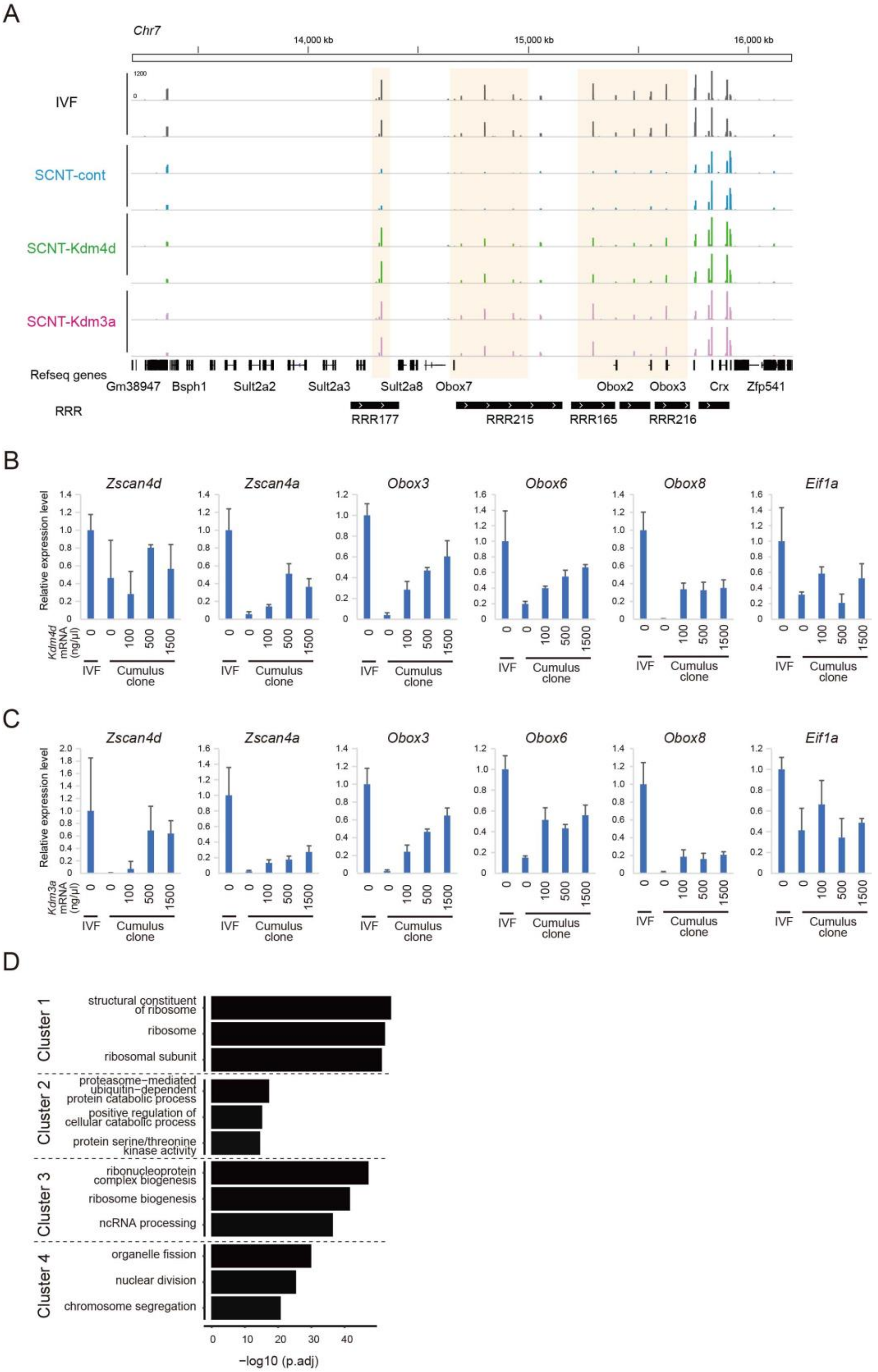

Figure S1. Effect of Kdm3a on the transcriptome of SCNT embryos (related to Figure 2)

(A) A genome browser view of RNA-seq data at the *Obox* cluster on chromosome 7 containing *Kdm3a/4d*-responsive genes (yellow box).

(B) Gene expression levels of ZGA genes in the *Kdm4d* -injected SCNT embryos examined by RT-qPCR. The expression levels were normalized to external control RNA. The value in IVF was set as 1.0.

(C) Gene expression levels of ZGA genes in the *Kdm3a* -injected SCNT embryos examined by RT-qPCR. The expression levels were normalized to external control RNA. The value in IVF was set as 1.0.

(D) Gene ontology analysis of the four clusters classified in Figure 2D.

Figure S2

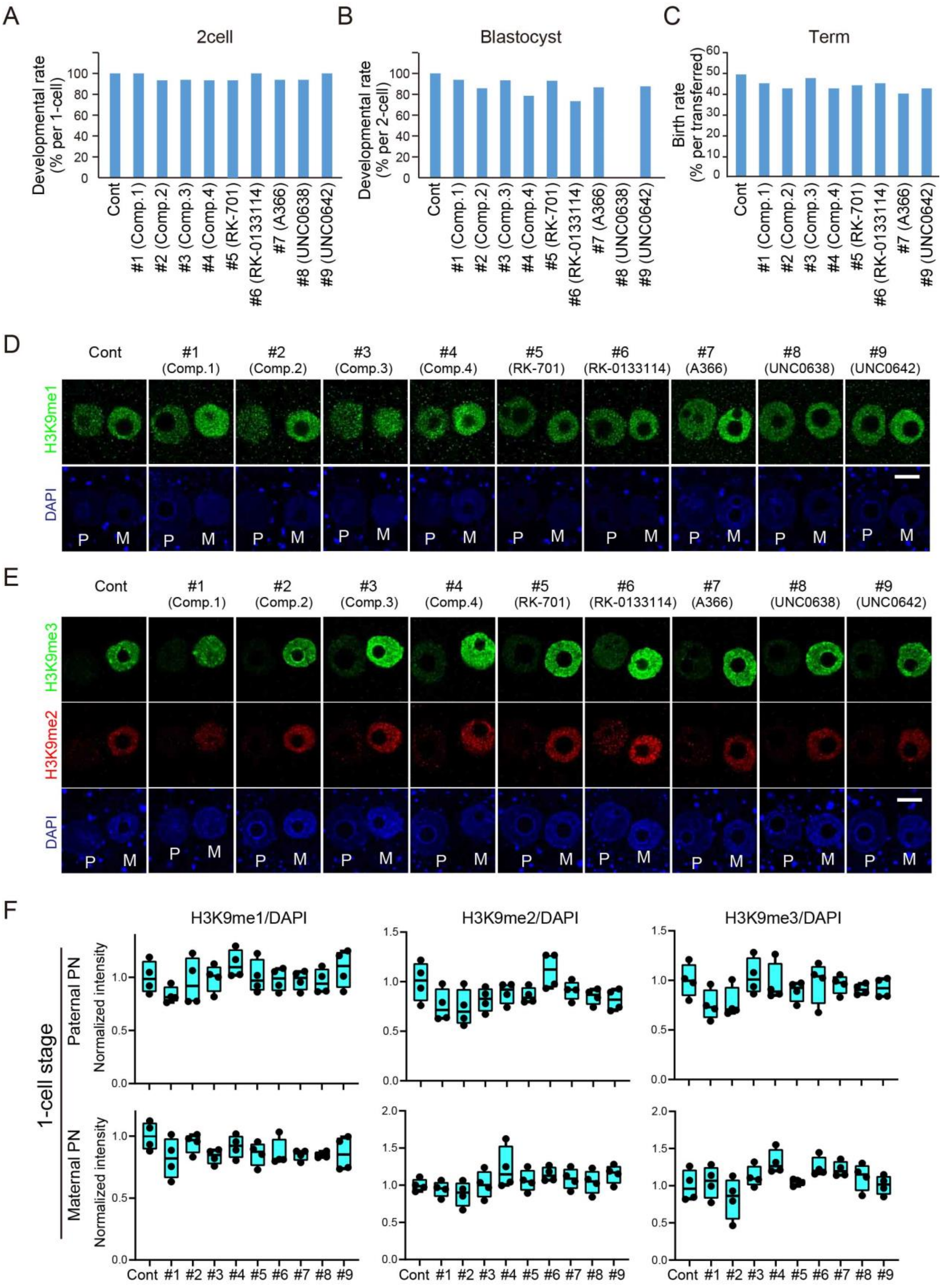
A
2cell
Developmental rate (% per 1-cell)
0
20
40
60
80
100
Cont
#1 (Comp.1)
#2 (Comp.2)
#3 (Comp.3)
#4 (Comp.4)
#5 (RK-701)
#6 (RK-0133114)
#7 (A366)
#8 (UNC0638)
#9 (UNC0642)
B
Blastocyst
Developmental rate (% per 2-cell)
C
Term
Birth rate (% per transferred)
0
10
20
30
40
50
60
D
Cont
#1 (Comp.1)
#2 (Comp.2)
#3 (Comp.3)
#4 (Comp.4)
#5 (RK-701)
#6 (RK-0133114)
#7 (A366)
#8 (UNC0638)
#9 (UNC0642)
H3K9me1
DAPI
P
M
E
H3K9me3
H3K9me2
DAPI
F
H3K9me1/DAPI
H3K9me2/DAPI
H3K9me3/DAPI
1-cell stage
Paternal PN
Maternal PN
Normalized intensity
0.0
0.5
1.0
1.5
2.0
Cont #1 #2 #3 #4 #5 #6 #7 #8 #9

Figure S2. Effects of G9a inhibitors on the development and H3K9 methylation levels of IVF embryos (related to Figure 4)

(A, B) Bar graphs showing the developmental rate to the two-cell stage (A), and the blastocyst stage (B). Embryos were treated with the G9a inhibitors indicated at 1 μM concentration from 5 hpi throughout the cultivation period. RK-0133114 is the inactive *R*-enantiomer form of RK-701. All G9ai used in this figure are listed in Table S6.

(C) Bar graphs showing the developmental rate to term. Embryos were treated with the G9a inhibitors indicated at 1 μM concentration from 5 hpi until 24hpi. The embryos were transferred to the oviduct of pseudopregnant females at the 2-cell stage.

(D) Representative images of IVF embryos at the one-cell stage stained with anti-H3K9me1 antibody and DAPI. P, paternal pronucleus. M, maternal pronucleus. Scale bar represents 10 μm.

(E) Representative images of IVF embryos at the one-cell stage stained with anti-H3K9me2 and anti-H3K9me3 antibodies and DAPI. P, paternal pronucleus. M, maternal pronucleus. Scale bar represents 10 μm.

(F) Box plots comparing the relative intensities of H3K9me1, H3K9me2, and H3K9me3 in the IVF one-cell stage embryos. Each signal intensity was normalized to the DAPI signal levels. Four to five embryos were analyzed for each condition.

Figure S3

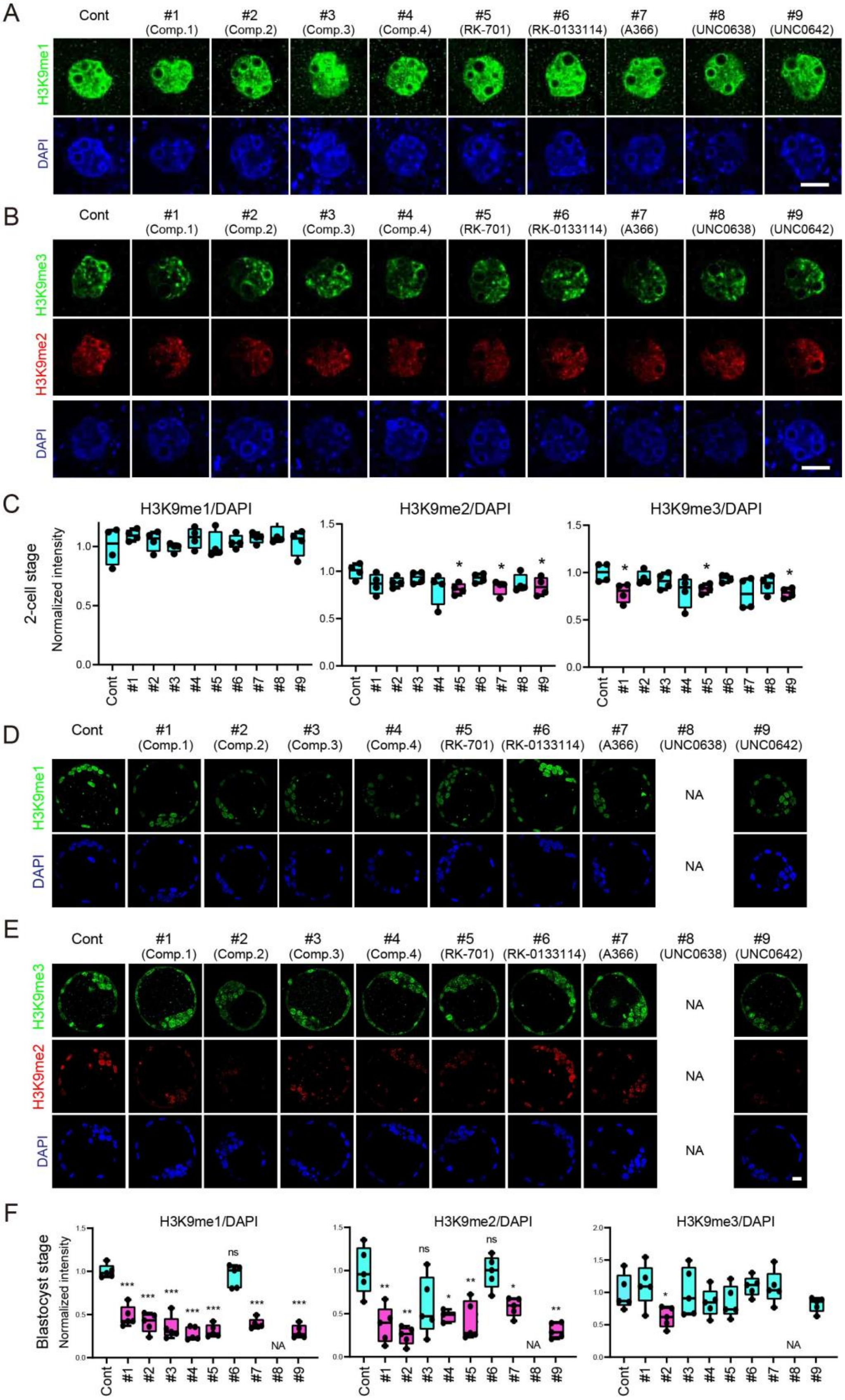
A
B
C
D
E
F
Cont
#1 (Comp.1)
#2 (Comp.2)
#3 (Comp.3)
#4 (Comp.4)
#5 (RK-701)
#6 (RK-0133114)
#7 (A366)
#8 (UNC0638)
#9 (UNC0642)
H3K9me1
H3K9me2
H3K9me3
DAPI
NA
H3K9me1/DAPI
H3K9me2/DAPI
H3K9me3/DAPI
2-cell stage
Blastocyst stage
Normalized intensity
ns

Figure S3. Effects of G9a inhibitors on the H3K9 methylation levels of IVF embryos (related to Figure 4)

(A) Representative images of IVF embryos at the two-cell stage stained with anti-H3K9me1 antibody and DAPI. Scale bar represents 10 μm.

(B) Representative images of IVF embryos at the two-cell stage stained with anti-H3K9me2 and anti-H3K9me3 antibodies and DAPI. Scale bar represents 10 μm.

(C) Box plots comparing the relative intensities of H3K9me1, H3K9me2, and H3K9me3 in the IVF two-cell stage embryos. Each signal intensity was normalized to the DAPI signal levels. Four to five embryos were analyzed for each condition. *$P < 0.05$ compared with the control.

(D) Representative images of IVF embryos at the blastocyst stage stained with anti-H3K9me1 antibody and DAPI. Scale bar represents 10 μm.

(E) Representative images of IVF embryos at the blastocyst stage stained with anti-H3K9me2 and anti-H3K9me3 antibodies and DAPI. Scale bar represents 10 μm.

(F) Box plots comparing the relative intensities of H3K9me1, H3K9me2, and H3K9me3 in the IVF blastocysts. Each signal intensity was normalized to the DAPI signal levels. Five embryos were analyzed for each condition. *$P < 0.05$, **$P < 0.01$ ***$P < 0.001$ compared with the control.

Figure S4

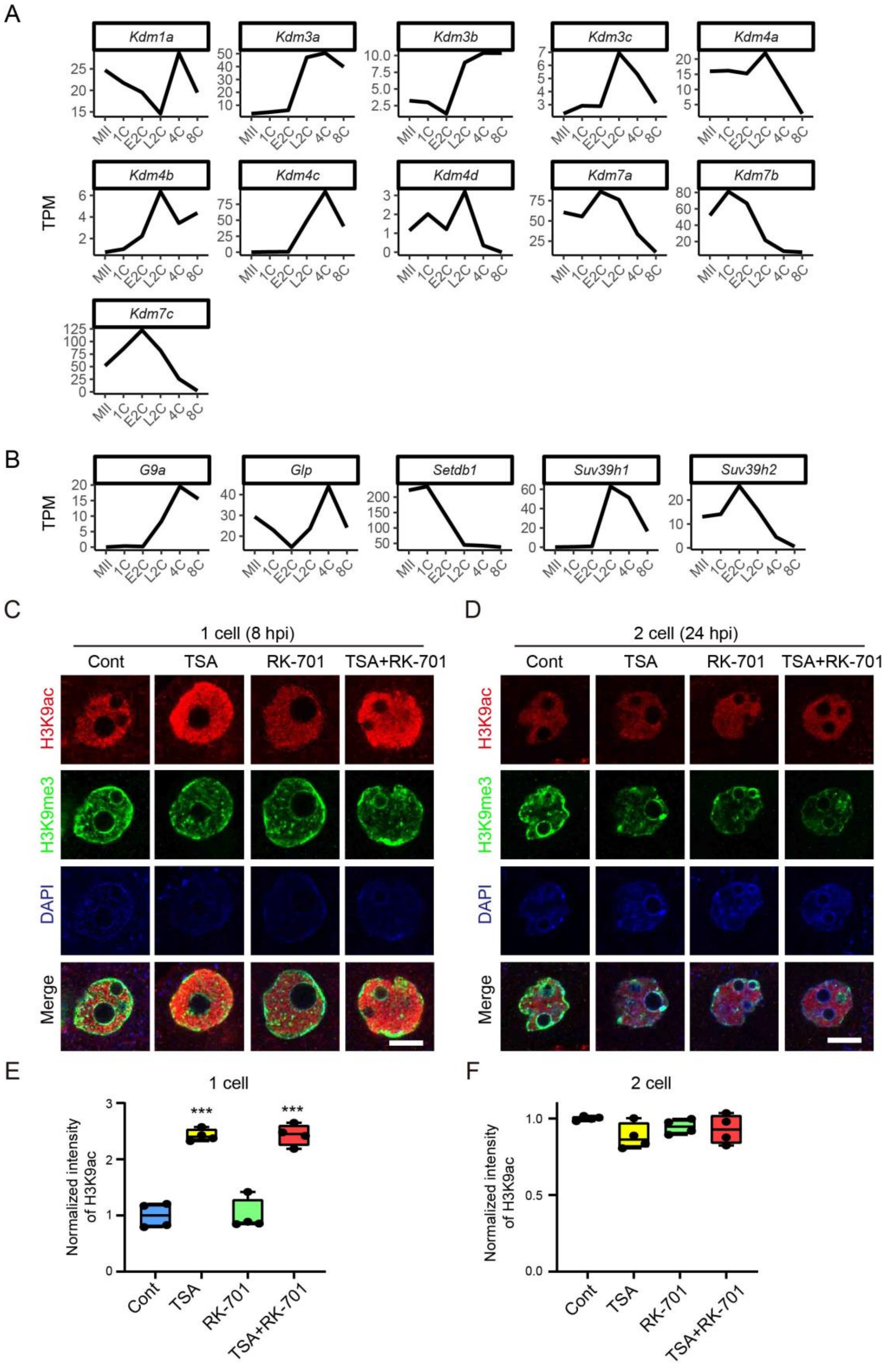
A
Kdm1a
Kdm3a
Kdm3b
Kdm3c
Kdm4a
Kdm4b
Kdm4c
Kdm4d
Kdm7a
Kdm7b
Kdm7c
TPM
MII 1C E2C L2C 4C 8C
B
G9a
Glp
Setdb1
Suv39h1
Suv39h2
TPM
C
1 cell (8 hpi)
Cont TSA RK-701 TSA+RK-701
H3K9ac
H3K9me3
DAPI
Merge
D
2 cell (24 hpi)
Cont TSA RK-701 TSA+RK-701
E
1 cell
Normalized intensity of H3K9ac
***
Cont TSA RK-701 TSA+RK-701
F
2 cell
Normalized intensity of H3K9ac
Cont TSA RK-701 TSA+RK-701

Figure S4. Gene expression levels of histone methyltransferases and demethylases for H3K9me2 in the mouse preimplantation embryos and H3K9 acetylation levels in the SCNT embryos (related to Figure 4)

(A) Line plots showing the gene expression levels of histone demethylases with H3K9 demethylation activities in the mouse preimplantation embryos. The gene expression data was obtained from GSE71434 (Zhang et al., 2016).

(B) Line plots showing the gene expression levels of histone methyltransferases for H3K9 deposition in the mouse preimplantation embryos. The gene expression data was obtained from GSE71434 (Zhang et al., 2016).

(C) Representative images of SCNT embryos at the one-cell stage stained with anti-H3K9ac and anti-H3K9me3 antibodies and DAPI. Scale bar represents 10 μm.

(D) Representative images of SCNT embryos at the two-cell stage stained with anti-H3K9ac and anti-H3K9me3 antibodies and DAPI. Scale bar represents 10 μm.

(E) Box plots comparing the normalized intensities of H3K9ac in the SCNT embryos at the one-cell stage. Each signal intensity was normalized to the DAPI signal levels. Four embryos were analyzed for each condition. ***$P < 0.001$ compared with the control.

(F) Box plots comparing the normalized intensities of H3K9ac in the SCNT embryos at the two-cell stage. Each signal intensity was normalized to the DAPI signal levels. Four embryos were analyzed for each condition.

Figure S5

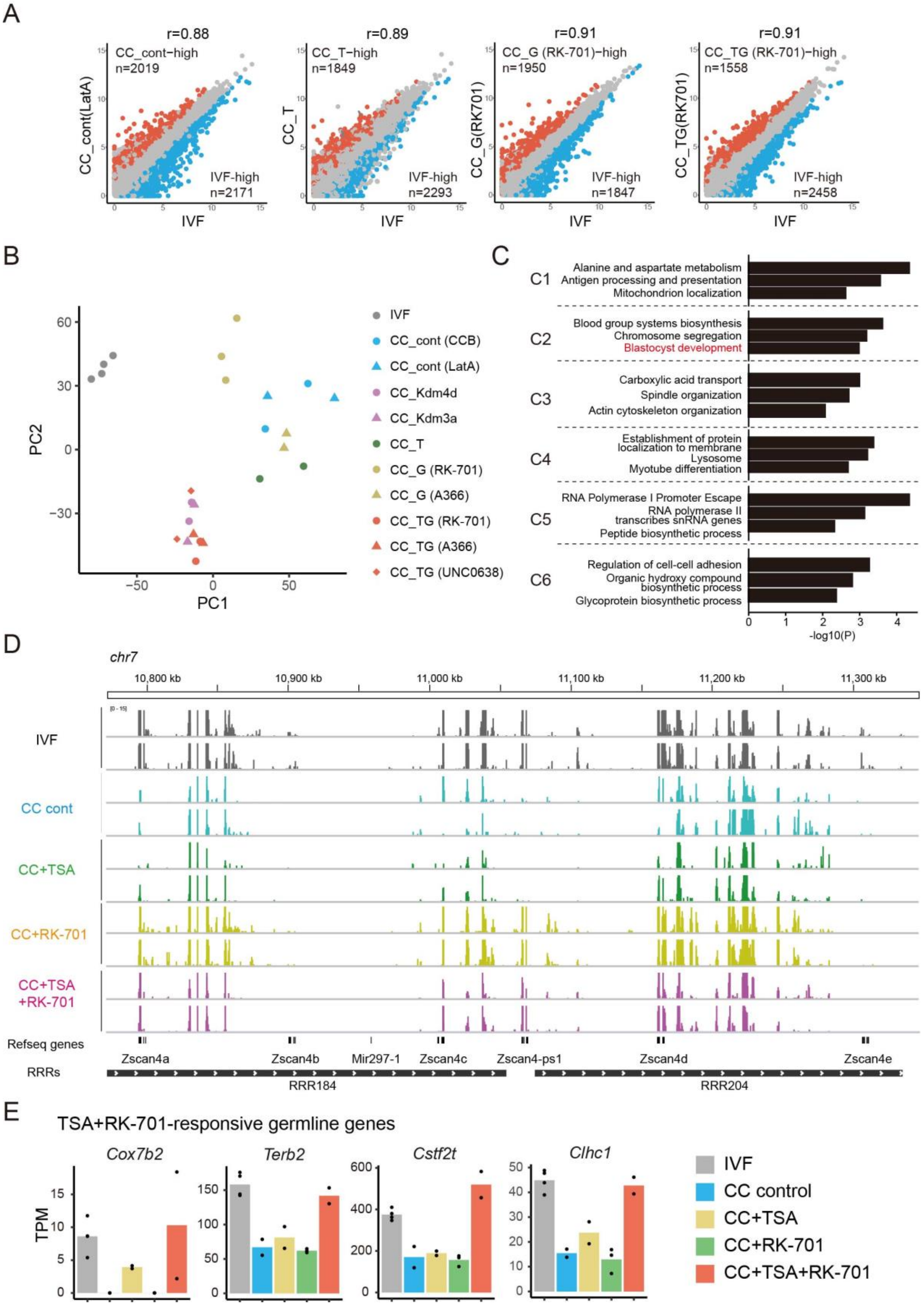
A
r=0.88
CC_cont-high
n=2019
CC_cont(LatA)
IVF-high
n=2171
IVF
r=0.89
CC_T-high
n=1849
CC_T
IVF-high
n=2293
IVF
r=0.91
CC_G (RK-701)-high
n=1950
CC_G(RK701)
IVF-high
n=1847
IVF
r=0.91
CC_TG (RK-701)-high
n=1558
CC_TG(RK701)
IVF-high
n=2458
IVF
B
PC2
PC1
IVF
CC_cont (CCB)
CC_cont (LatA)
CC_Kdm4d
CC_Kdm3a
CC_T
CC_G (RK-701)
CC_G (A366)
CC_TG (RK-701)
CC_TG (A366)
CC_TG (UNC0638)
C
C1
Alanine and aspartate metabolism
Antigen processing and presentation
Mitochondrion localization
C2
Blood group systems biosynthesis
Chromosome segregation
Blastocyst development
C3
Carboxylic acid transport
Spindle organization
Actin cytoskeleton organization
C4
Establishment of protein localization to membrane
Lysosome
Myotube differentiation
C5
RNA Polymerase I Promoter Escape
RNA polymerase II transcribes snRNA genes
Peptide biosynthetic process
C6
Regulation of cell-cell adhesion
Organic hydroxy compound biosynthetic process
Glycoprotein biosynthetic process
-log10(P)
D
chr7
10,800 kb
10,900 kb
11,000 kb
11,100 kb
11,200 kb
11,300 kb
IVF
CC cont
CC+TSA
CC+RK-701
CC+TSA +RK-701
Refseq genes
Zscan4a
Zscan4b
Mir297-1
Zscan4c
Zscan4-ps1
Zscan4d
Zscan4e
RRRs
RRR184
RRR204
E
TSA+RK-701-responsive germline genes
Cox7b2
Terb2
Cstf2t
Clhc1
TPM
IVF
CC control
CC+TSA
CC+RK-701
CC+TSA+RK-701

Figure S5. Effect of G9ai on the transcriptome of SCNT embryos at the two-cell stage (related to Figure 5)

(A) Scatterplots comparing the gene expression levels of IVF and SCNT embryos at the two-cell stage. The genes with significant differences (FC > 2) are colored red or blue.

(B) PCA plot of the transcriptome derived from the two-cell stage embryos. Genes with TPM > 1 on average among all samples were used. Each dot represents a single sample. Note that G9ai and TSA synergistically converted the transcriptome of SCNT embryos and that three different G9ai-treated samples co-treated with TSA (CC_TG (RK-701), CC_TG (A366), and CC_TG (UNC0638)) were closely clustered together with *Kdm3a*- and *Kdm4d*-injected embryos.

(C) Gene ontology analysis of the six clusters (C1: Cluster 1) classified in Figure 5B. Note that the "Blastocyst development" term was enriched in C2.

(D) A genome browser view of RNA-seq data at the *Zscan4* cluster on chromosome 7.

(E) Bar graphs showing the expression levels of TSA+RK-701-responsive germline genes.

Figure S6

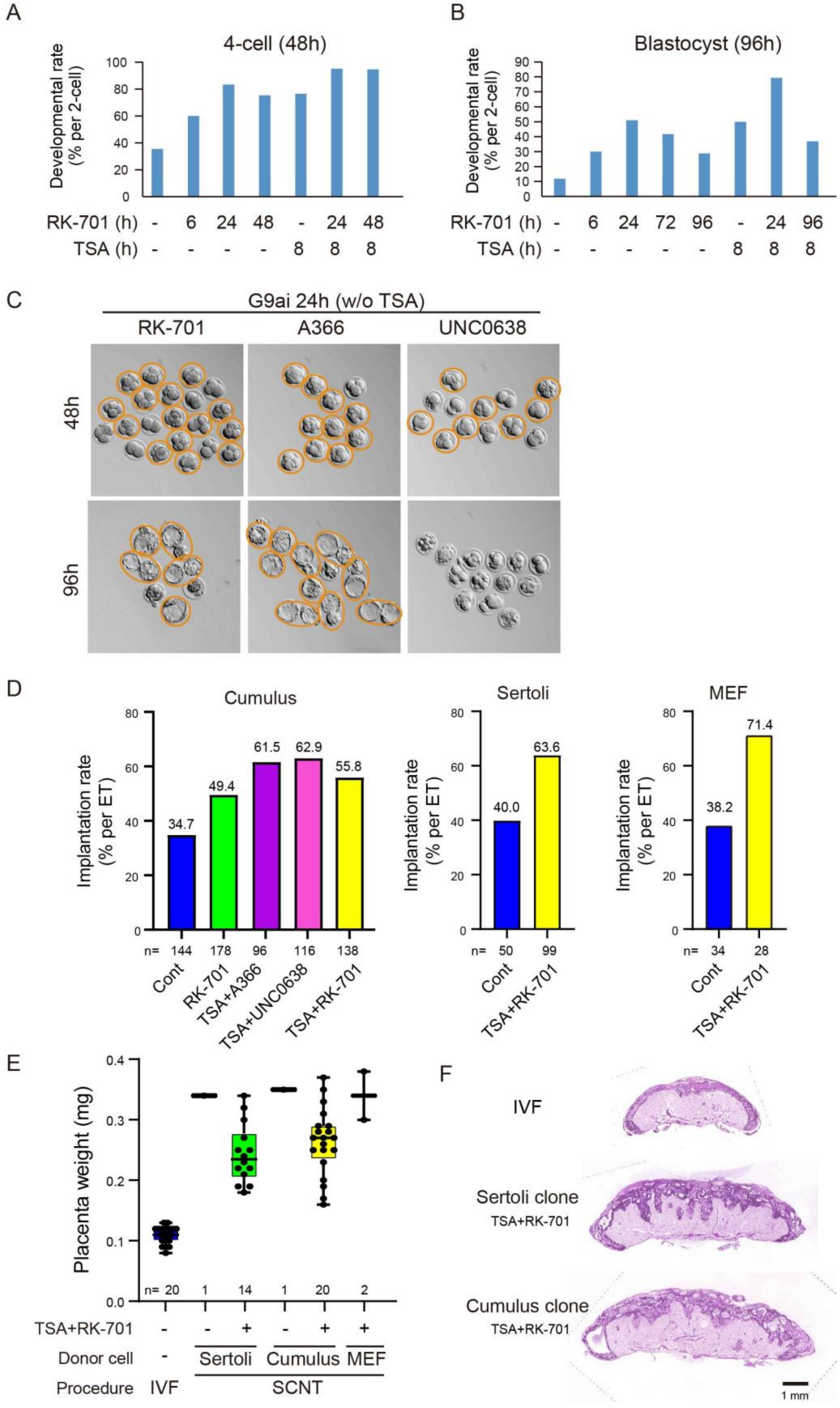
A
4-cell (48h)
Developmental rate (% per 2-cell)
100
80
60
40
20
0
RK-701 (h) - 6 24 48 - 24 48
TSA (h) - - - - 8 8 8
B
Blastocyst (96h)
Developmental rate (% per 2-cell)
90 80 70 60 50 40 30 20 10 0
RK-701 (h) - 6 24 72 96 - 24 96
TSA (h) - - - - - 8 8 8
C
G9ai 24h (w/o TSA)
RK-701
A366
UNC0638
48h
96h
D
Cumulus
Implantation rate (% per ET)
80 60 40 20 0
34.7 49.4 61.5 62.9 55.8
n= 144 178 96 116 138
Cont RK-701 TSA+A366 TSA+UNC0638 TSA+RK-701
Sertoli
40.0 63.6
n= 50 99
Cont TSA+RK-701
MEF
38.2 71.4
n= 34 28
Cont TSA+RK-701
E
Placenta weight (mg)
0.4 0.3 0.2 0.1 0.0
n= 20 1 14 1 20 2
TSA+RK-701 - - + - + +
Donor cell - Sertoli Cumulus MEF
Procedure IVF SCNT
F
IVF
Sertoli clone
TSA+RK-701
Cumulus clone
TSA+RK-701
1 mm

Figure S6. Effects of G9ai on the embryonic development of SCNT embryos (related to Figure 6)

(A, B) Bar graph showing the developmental rate of SCNT embryos derived from cumulus cells at the four-cell stage (A) and the blastocyst stage (B).

(C) Representative images of SCNT embryos derived from cumulus cells at 48 and 96 h of in vitro culture. Normally developing embryos are marked with orange circles. Scale bar, 100 μm.

(D) Bar graphs showing the implantation rate of SCNT embryos examined by the caesarean section on E19.5.

(E) Box plots showing the weight of placentae examined by the caesarean section on E19.5. The whiskers represent the maximum and minimum, and n represents the number of placentae examined.

(F) Representative images of histological sections of a term placenta stained with periodic acid-Schiff (PAS). Microscopic images for each sample were combined into a single panel by adjusting the scale. Note that the PAS-positive spongiotrophoblast layer invaded into the labyrinthine layer in the SCNT placenta even with TSA and RK-701 co-treatment. Scale bar, 1 mm.

Supplemental Tables

Table S1. Gene expression levels in all samples analyzed in this study (related to Figures 2 and 5; see Excel file)

Table S2. Preimplantation development of SCNT embryos (related to Figures 3 and 6)

Donor cell		Name of sample	Actin polymer-ization inhibitor	mRNA injected	G9a inhibitor (1 µM, 24 h)	HDAC inhibitor (25 nM, 8 h)	No. of 1-cell embryos	No. of cleaved embryos (% per 1-cell)	No. of 4-cell embryos (% per cleaved)	No. of 8-cell or morula embryos (% per cleaved)	No. of blastocysts (% per cleaved)
Cell type	Sex										
Cumulus	Female	SCNT_Cont (CCB)	CCB	–	–	–	54	50 (93)	15 (30)	9 (18)	7 (14)
		SCNT_Kdm3a	CCB	*Kdm3a*	–	–	64	60 (94)	56 (93)	48 (80)	42 (70)
		SCNT_Kdm4d	CCB	*Kdm4d*	–	–	60	57 (95)	54 (95)	50 (88)	46 (81)
		CC_Cont (LatA)	LatA	–	–	–	53	49 (92)	17 (35)	9 (18)	8 (16)
		CC_T	LatA	–	–	TSA	58	55 (95)	42 (76)	33 (60)	29 (53)
		CC_G (RK-701)	LatA	–	RK-701	–	63	59 (94)	46 (78)	36 (61)	26 (44)
		CC_TG (RK-701)	LatA	–	RK-701	TSA	78	75 (96)	71 (95)	62 (83)	60 (80)
		CC_TG (A366)	LatA	–	A366	TSA	72	70 (97)	69 (99)	57 (81)	55 (79)
		CC_TG (UNC0638)	LatA	–	UNC0638	TSA	79	74 (94)	72 (97)	67 (91)	63 (85)
Sertoli	Male	SC_Cont	LatA	–	–	–	89	79 (89)	26 (33)	20 (25)	12 (15)
		SC_TG (RK-701)	LatA	–	RK-701	TSA	105	92 (88)	87 (95)	83 (90)	78 (85)
MEF	Male	MEFC_Cont	LatA	–	–	–	55	49 (89)	10 (20)	7 (14)	5 (10)
		MEFC_TG (RK-701)	LatA	–	RK-701	TSA	53	47 (89)	43 (91)	41 (87)	39 (83)

The concentration of injected *Kdm3a* and *Kdm4d* mRNA was 1500 ng/µl. CCB, cytochalasin B. LatA, latrunculin A. HDAC, histone deacetylase. TSA, trichostatin A.

Table S3. Postimplantation development of IVF embryos treated with G9ai (related to Figure 4)

Name of sample	G9a inhibitor (1 μM, 24 h)	No. of 2-cell embryos transferred	No. of recipient females	No. of implanted (% per ET)	No. of pups at birth (% per ET)	Body weight at birth (g ± SD)	Placenta weight at birth (g ± SD)
Control	–	65	4	41 (63.1)	32 (49.2)	1.52 ± 0.13	0.1 ± 0.02
#1	Compound 1	40	2	23 (57.5)	18 (45.0)	1.47 ± 0.12	0.11 ± 0.02
#2	Compound 2	40	2	25 (62.5)	17 (42.5)	1.54 ± 0.12	0.1 ± 0.01
#3	Compound 3	40	2	23 (57.5)	19 (47.5)	1.5 ± 0.11	0.1 ± 0.01
#4	Compound 4	40	2	24 (60.0)	17 (42.5)	1.54 ± 0.11	0.1 ± 0.01
#5	RK-701	50	3	31 (62.0)	22 (44.0)	1.5 ± 0.1	0.11 ± 0.01
#6	RK-0133114	40	2	25 (62.5)	18 (45.0)	1.48 ± 0.13	0.1 ± 0.01
#7	A366	40	2	26 (65.0)	16 (40.0)	1.5 ± 0.1	0.11 ± 0.01
#9	UNC0642	40	2	24 (60.0)	17 (42.5)	1.51 ± 0.08	0.11 ± 0.01

All embryos were generated by IVF using BDF1 females and males. ET, embryo transfer.

Table S4. List of 879 RRGs (related to Figure 5; see Excel file)

Table S5. Postimplantation development of SCNT embryos (related to Figure 6)

Donor cell		Name of sample	G9a inhibitor (1 μM, 24 h)	HDAC inhibitor (25 nM, 8 h)	No. of 2-cell embryos transferred	No. of implanted (% per ET)	No. of pups at birth (% per ET)	No. of pups survived to adults (% per birth)	Body weight at birth (g ± SD)	Placenta weight at birth (g ± SD)
Cell type	Sex									
Cumulus	Female	CC_Cont	–	–	144	50 (34.7)	1 (0.7)	1 (100)	1.65	0.35
		CC_RK-701	RK-701	–	178	88 (49.4)	7 (3.9)	6 (85.7)	1.57 ± 0.13	0.29 ± 0.06
		CC_TSA+A366	A366	TSA	96	59 (61.5)	11 (11.5)	10 (90.9)	1.42 ± 0.14	0.27 ± 0.05
		CC_TSA+UNC0638	UNC0638	TSA	116	73 (62.9)	14 (12.1)	12 (85.7)	1.42 ± 0.11	0.27 ± 0.04
		CC_TSA+RK-701	RK-701	TSA	138	77 (55.8)	20 (14.5)	18 (90)	1.48 ± 0.17	0.26 ± 0.06
Sertoli	Male	SC_Cont	–	–	50	20 (40.0)	1 (1.7)	1 (100)	0.34	0.34
		SC_TSA+RK-701	RK-701	TSA	99	63 (63.6)	14 (14.1)	14 (100)	1.43 ± 0.10	0.24 ± 0.05
MEF	Male	MEFC_Cont	–	–	34	13 (38.2)	0 (0.0)	N/A	N/A	N/A
		MEFC_TSA+RK-701	RK-701	TSA	28	20 (71.4)	2 (7.1)	1 (50.0)	1.69 ± 0.06	0.34 ± 0.06

The concentration of injected *Kdm3a* and *Kdm4d* mRNA was 1500 ng/μl. HDAC, histone deacetylase. TSA, trichostatin A. ET, embryo transfer. N/A, not applicable.

Table S6. List of G9a inhibitors used in this study

Name of inhibitor	IC50 to G9a (nM)	Company	Catalog #	Notes	Reference/Patent #
RK-701	23–27	-	NA		(Nishigaya et al., 2023) (Takase et al., 2023)
RK-0133114	3,700	-	NA	*R*-enantiomer of RK-701	(Nishigaya et al., 2023) (Takase et al., 2023)
Compound 1	21	-	#13-1 in patent		#WO2021106988A1
Compound 2	2.0	-	#6-89 in patent		#WO2021106988A1
Compound 3	24	-	#6-32 in patent		#WO2021106988A1
Compound 4	2.9	-	#1-67 in patent		#WO2021106988A1
A366	3.3	Merck	#SML1410		(Sweis et al., 2014)
UNC0638	< 15	Merck	#U4885		(Vedadi et al., 2011)
UNC0642	< 2.5	Merck	#SML1037		(Liu et al., 2013)

Supplemental References

Liu, F., Barsyte-Lovejoy, D., Li, F., Xiong, Y., Korboukh, V., Huang, X.P., Allali-Hassani, A., Janzen, W.P., Roth, B.L., Frye, S. V., et al. (2013). Discovery of an in vivo chemical probe of the lysine methyltransferases G9a and GLP. J. Med. Chem. *56*, 8931–8942. 10.1021/JM401480R.

Matoba, S., Inoue, K., Kohda, T., Sugimoto, M., Mizutani, E., Ogonuki, N., Nakamura, T., Abe, K., Nakano, T., Ishino, F., et al. (2011). RNAi-mediated knockdown of Xist can rescue the impaired postimplantation development of cloned mouse embryos. Proc. Natl. Acad. Sci. U. S. A. *108*, 20621–20626. 10.1073/pnas.1112664108.

Matoba, S., Wang, H., Jiang, L., Lu, F., Iwabuchi, K.A., Wu, X., Inoue, K., Yang, L., Press, W., Lee, J.T., et al. (2018). Loss of H3K27me3 imprinting in somatic cell nuclear transfer embryos disrupts post-implantation development. Cell Stem Cell *23*, 343–354. 10.1016/j.stem.2018.06.008.

Nishigaya, Y., Takase, S., Sumiya, T., Kikuzato, K., Sato, T., Niwa, H., Sato, S., Nakata, A., Sonoda, T., Hashimoto, N., et al. (2023). Discovery of novel substrate-competitive lysine methyltransferase G9a inhibitors as anticancer agents. J. Med. Chem. *66*, 4059–4085. 10.1021/acs.jmedchem.2c02059.

Sweis, R.F., Pliushchev, M., Brown, P.J., Guo, J., Li, F., Maag, D., Petros, A.M., Soni, N.B., Tse, C., Vedadi, M., et al. (2014). Discovery and development of potent and selective inhibitors of histone methyltransferase G9a. ACS Med. Chem. Lett. *5*, 205–209. 10.1021/ml400496h.

Takase, S., Hiroyama, T., Shirai, F., Maemoto, Y., Nakata, A., Arata, M., Matsuoka, S., Sonoda, T., Niwa, H., Sato, S., et al. (2023). A specific G9a inhibitor unveils BGLT3 lncRNA as a universal mediator of chemically induced fetal globin gene expression. Nat. Commun. *14*, 23. 10.1038/s41467-022-35404-0.

Vedadi, M., Barsyte-Lovejoy, D., Liu, F., Rival-Gervier, S., Allali-Hassani, A., Labrie, V., Wigle, T.J., DiMaggio, P.A., Wasney, G.A., Siarheyeva, A., et al. (2011). A chemical probe selectively inhibits G9a and GLP methyltransferase activity in cells. Nat. Chem. Biol. *7*, 566–574. 10.1038/nchembio.599.

Zhang, B., Zheng, H., Huang, B., Li, W., Xiang, Y., Peng, X., Ming, J., Wu, X., Zhang, Y., Xu, Q., et al. (2016). Allelic reprogramming of the histone modification H3K4me3 in early mammalian development. Nature *537*, 553–557. 10.1038/nature19361.
