# Supplementary material for: Reduction of H3K9 methylation by G9a inhibitors improves the development of mouse SCNT embryos
Source: Stem Cell Reports. 2024 May 9;19(6):906–21. doi: 10.1016/j.stemcr.2024.04.003 (PMC11390627; doi:10.1016/j.stemcr.2024.04.003)
Supplement: Document S2. Article plus supplemental information [file mmc4.pdf]

Stem Cell Reports
Resource
OPEN ACCESS

Reduction of H3K9 methylation by G9a inhibitors improves the development of mouse SCNT embryos

Shogo Matoba,[1,2,11,*] Daiki Shikata,[1,3] Fumiyuki Shirai,[4] Takaki Tatebe,[1,3] Michiko Hirose,[1] Akiko Nakata,[5] Naomi Watanabe,[1,3] Ayumi Hasegawa,[1] Akihiro Ito,[6,7] Minoru Yoshida,[5,7,8] and Atsuo Ogura[1,3,9,10,*]
[1]Bioresource Engineering Division, Bioresource Research Center, RIKEN, Tsukuba, Ibaraki 305-0074, Japan
[2]Cooperative Division of Veterinary Sciences, Tokyo University of Agriculture and Technology, Fuchu, Tokyo 183-8509, Japan
[3]Graduate School of Life and Environmental Sciences, University of Tsukuba, Tsukuba, Ibaraki 305-8572, Japan
[4]Drug Discovery Chemistry Platform Unit, RIKEN Center for Sustainable Resource Science, Wako, Saitama 351-0198, Japan
[5]Drug Discovery Seed Compounds Exploratory Unit, RIKEN Center for Sustainable Resource Science, Wako, Saitama 351-0198, Japan
[6]Laboratory of Cell Signaling, School of Life Sciences, Tokyo University of Pharmacy and Life Sciences, Hachioji, Tokyo 192-0392, Japan
[7]Chemical Genomics Research Group, RIKEN Center for Sustainable Resource Science, Wako, Saitama 351-0198, Japan
[8]Office of University Professors, The University of Tokyo, Bunkyo-ku, Tokyo 113-8657, Japan
[9]The Center for Disease Biology and Integrative Medicine, Faculty of Medicine, University of Tokyo, Tokyo 113-0033, Japan
[10]Bioresource Engineering Laboratory, RIKEN Cluster for Pioneering Research, Wako, Saitama 351-0198, Japan
[11]Lead contact
*Correspondence: shogo.matoba@riken.jp (S.M.), atsuo.ogura@riken.jp (A.O.)
https://doi.org/10.1016/j.stemcr.2024.04.003

SUMMARY

Removal of somatic histone H3 lysine 9 trimethylation (H3K9me3) from the embryonic genome can improve the efficiency of mammalian cloning using somatic cell nuclear transfer (SCNT). However, this strategy involves the injection of histone demethylase mRNA into embryos, which is limiting because of its invasive and labor-consuming nature. Here, we report that treatment with an inhibitor of G9a (G9ai), the major histone methyltransferase that introduces H3K9me1/2 in mammals, greatly improved the development of mouse SCNT embryos. Intriguingly, G9ai caused an immediate reduction of H3K9me1/2, a secondary loss of H3K9me3 in SCNT embryos, and increased the birth rate of cloned pups about 5-fold (up to 3.9%). G9ai combined with the histone deacetylase inhibitor trichostatin A further improved this rate to 14.5%. Mechanistically, G9ai and TSA synergistically enhanced H3K9me3 demethylation and boosted zygotic genome activation. Thus, we established an easy, highly effective SCNT protocol that would enhance future cloning research and applications.

INTRODUCTION

Somatic cell nuclear transfer (SCNT) enables the generation of individuals genetically identical to the donor somatic cell (so-called cloning) and has a wide variety of potential applications including biomedicine, bioindustry, and preservation of endangered species (Matoba and Zhang, 2018). However, the very low efficiency of obtaining cloned individuals has hampered the practical use of SCNT.

We have previously reported that histone H3 lysine 9 trimethylation (H3K9me3) present in donor somatic cells inhibits zygotic genome activation (ZGA) involved in transcription at the one- to two-cell stages in the mouse SCNT model (Matoba et al., 2014). Importantly, this H3K9me3 barrier could be removed by microinjection of mRNA for the histone demethylase *Kdm4d* or *Kdm4b* at the one-cell stage, which has been reported to significantly improve the embryonic development of SCNT embryos (Liu et al., 2016; Matoba et al., 2014). This *Kdm4* method is effective in a variety of species, not just mice, and has contributed to the efficient establishment of human SCNT embryo-derived embryonic stem cells (Chung et al., 2015) and the first cloning in non-human primate macaques (Liu et al., 2018). However, the *Kdm4* mRNA injection method is invasive, can damage the embryos, is technically demanding, and has a low throughput because of the need for micromanipulation.

H3K9me3 is an epigenetic mark that accumulates in heterochromatin and is catalyzed predominantly by SETDB1 and SUV39H1/2 in mammals (Padeken et al., 2022). SUV39H1/2 appears to be responsible primarily for the formation of the H3K9me3 barrier of SCNT reprogramming, since the knockdown of *Suv39h1/2*, but not *Setdb1*, in donor mouse embryonic fibroblasts (MEFs) has been reported to reduce the H3K9me3 level and improve the development of cloned embryos derived from these donor cells (Matoba et al., 2014). Although inhibitors of SUV39H1/2, such as chaetocin, have been developed, their low selectivity and very narrow range between the efficacy and toxicity doses make the use of these SUV39H1/2 inhibitors difficult (Cherblanc et al., 2013; Iwasa et al., 2010; Zhang et al., 2018). Chaetocin has been tried as a means of improving the developmental efficiency of SCNT embryos in many animal species, but no clear improvements have been achieved (Jafarpour et al., 2020; Jeong et al., 2020).

Methylation of H3K9 involves stepwise processes. For example, in *Caenorhabditis elegans* embryos, MET-2 first

 © 2024 The Authors.
This is an open access article under the CC BY license (http://creativecommons.org/licenses/by/4.0/).

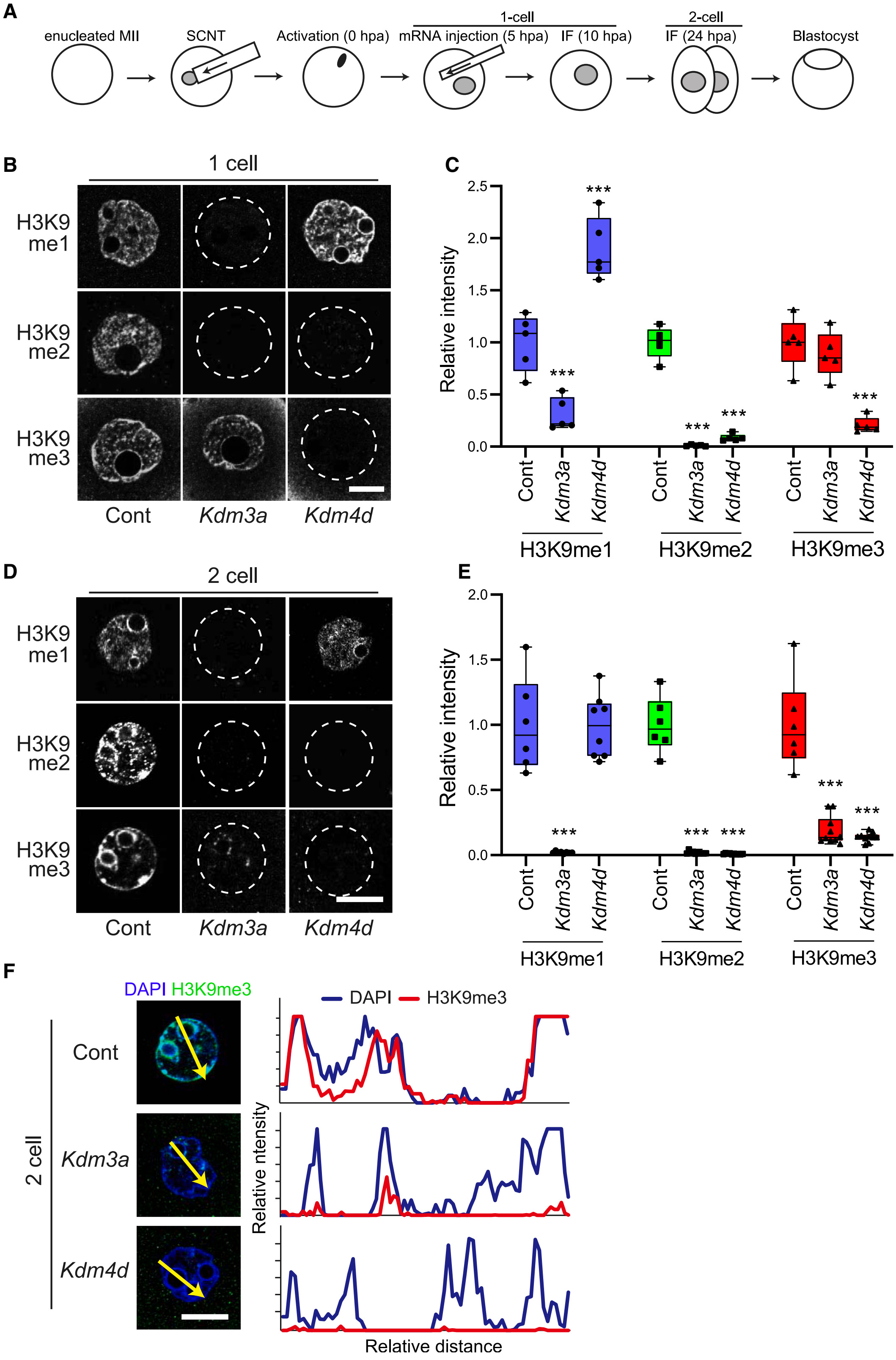

(legend on next page)

methylates unmethylated H3K9 into mono- (H3K9me1) and di-methylated H3K9 (H3K9me2), and SET-25 further methylates H3K9me1/2 to H3K9me3 to form heterochromatin (Towbin et al., 2012). Similarly, in mammals, PRDM3 and PRDM6 methylate unmethylated H3K9 in the cytoplasm to H3K9me1, which is then converted in the nucleus to H3K9me3 by SUV39H1/2 (Pinheiro et al., 2012). In the euchromatin regions, two methyltransferases, GLP/EHMT1 and G9a/EHMT2, are responsible for catalyzing the production of H3K9me1/2 (Tachibana et al., 2002; 2005), which can be methylated further by SUV39H1/2 to H3K9me3 (Bulut-Karslioglu et al., 2014; Peters et al., 2001). The aforementioned observations have raised the possibility that reduction of H3K9me3 could be achieved through the inhibition of its stepwise formation or maintenance processes.

In this study, we first found that targeted demethylation of H3K9me1/2 at the one-cell stage induced secondary loss of H3K9me3 at the two-cell stage in mouse SCNT embryos. We then used G9a/GLP inhibitors to inhibit the formation of H3K9me1/2 noninvasively, which resulted in a significant reduction of H3K9me3 in the SCNT embryos. We have optimized this approach by combining it with a histone deacetylase inhibitor (HDACi), which improved the cloning efficiency in mice. Our optimized non-invasive and genetic modification-free approach for improving SCNT provides a basis for future studies of and applications to animal cloning.

RESULTS

Demethylation of H3K9me1/2 by *Kdm3a* results in secondary erasure of H3K9me3 in the SCNT embryos

To explore the possible mechanisms through which the H3K9me3 level decreases in SCNT embryos, we searched for enzymes that could eliminate H3K9 methylation in the mouse model. Among the histone lysine demethylase (KDM) family members with histone H3K9-demethylating activity, KDM4D is known to target H3K9me2 and H3K9me3, and KDM3A to target H3K9me1 and H3K9me2 (Shi and Whetstine, 2007; Whetstine et al., 2006). To confirm the specificity of these demethylases, mouse SCNT embryos were injected with *Kdm4d* or *Kdm3a* mRNA at 5 h post activation (hpa), and the embryos were immunostained for three types of H3K9 methylation (H3K9me1, 2, or 3) at 10 hpa (Figure 1A). As previously reported (Matoba et al., 2014), *Kdm4d* mRNA injection resulted in almost complete demethylation of H3K9me2 and H3K9me3 (Figures 1B and 1C). H3K9me1 was significantly increased in these embryos, probably as a secondary product of the demethylation of H3K9me2/3 (Figures 1B and 1C). This reduced level of H3K9me2/3 was maintained in *Kdm4d* mRNA-injected embryos at the two-cell stage, when major ZGA begins, a finding that is consistent with our previous report (Matoba et al., 2014). By contrast, injection of *Kdm3a* resulted in the complete loss of H3K9me1 and H3K9me2 but not H3K9me3 at the one-cell stage in SCNT embryos (Figures 1B and 1C). Interestingly, analysis of these *Kdm3a*-injected embryos at the two-cell stage (24 hpa) showed that both H3K9me1/2 and H3K9me3 decreased greatly (Figures 1D and 1E). To carefully compare the localization of 4′,6-diamidino-2-phenylindole (DAPI) and H3K9me3, we analyzed their signal intensities by line profiling (Figure 1F). Although some of the nucleoli surrounding heterochromatin regions maintained a faint signal in *Kdm3a*-injected two-cell embryos, most DAPI-dense heterochromatic regions and non-DAPI-dense euchromatic regions lost H3K9me3 (Figure 1F). These results suggest that H3K9me3 could be eliminated secondary to the loss of H3K9me1/2.

The transcriptome of *Kdm3a*-treated SCNT embryos is similar to that of *Kdm4d*-treated SCNT embryos. To understand the transcriptional consequences of H3K9me1/2 demethylation by *Kdm3a*, we used RNA sequencing (RNA-seq) to compare the transcriptome of *in vitro* fertilized (IVF) embryos, control SCNT embryos, and SCNT embryos injected with *Kdm3a* (SCNT-*Kdm3a*) or *Kdm4d* mRNA

Figure 1. Removal of H3K9me1/2 by *Kdm3a* led to a secondary loss of H3K9me3 in mouse SCNT embryos
(A) Schematic illustration of the experimental approach. SCNT embryos were injected with either *Kdm3a* or *Kdm4d* mRNA at 6 hpi and stained for immunofluorescence at the one-cell (10 hpi) and two-cell (24 hpi) stages.
(B) Representative images of the nuclei of SCNT embryos immuno-stained using anti-H3K9me1, anti-H3K9me2, and anti-H3K9me3 antibodies at the one-cell stage. Scale bar, 5 μm.
(C) Boxplots showing the relative intensities of H3K9me1/2/3 levels in each sample at the one-cell stage. ***$p < 0.001$ compared with the control.
(D) Representative images of nuclei of SCNT embryos immuno-stained using anti-H3K9me1, anti-H3K9me2, and anti-H3K9me3 antibodies at the two-cell stage. Scale bar, 5 μm.
(E) Boxplots showing the relative intensities of H3K9me1/2/3 levels in each sample at the one-cell stage. ***$p < 0.001$ compared with the control.
(F) Line profiles (yellow arrows) of 4′,6-diamidino-2-phenylindole (DAPI) and H3K9me3 channels showing the relative intensities in the nucleus of the two-cell stage embryos. Scale bar, 5 μm.

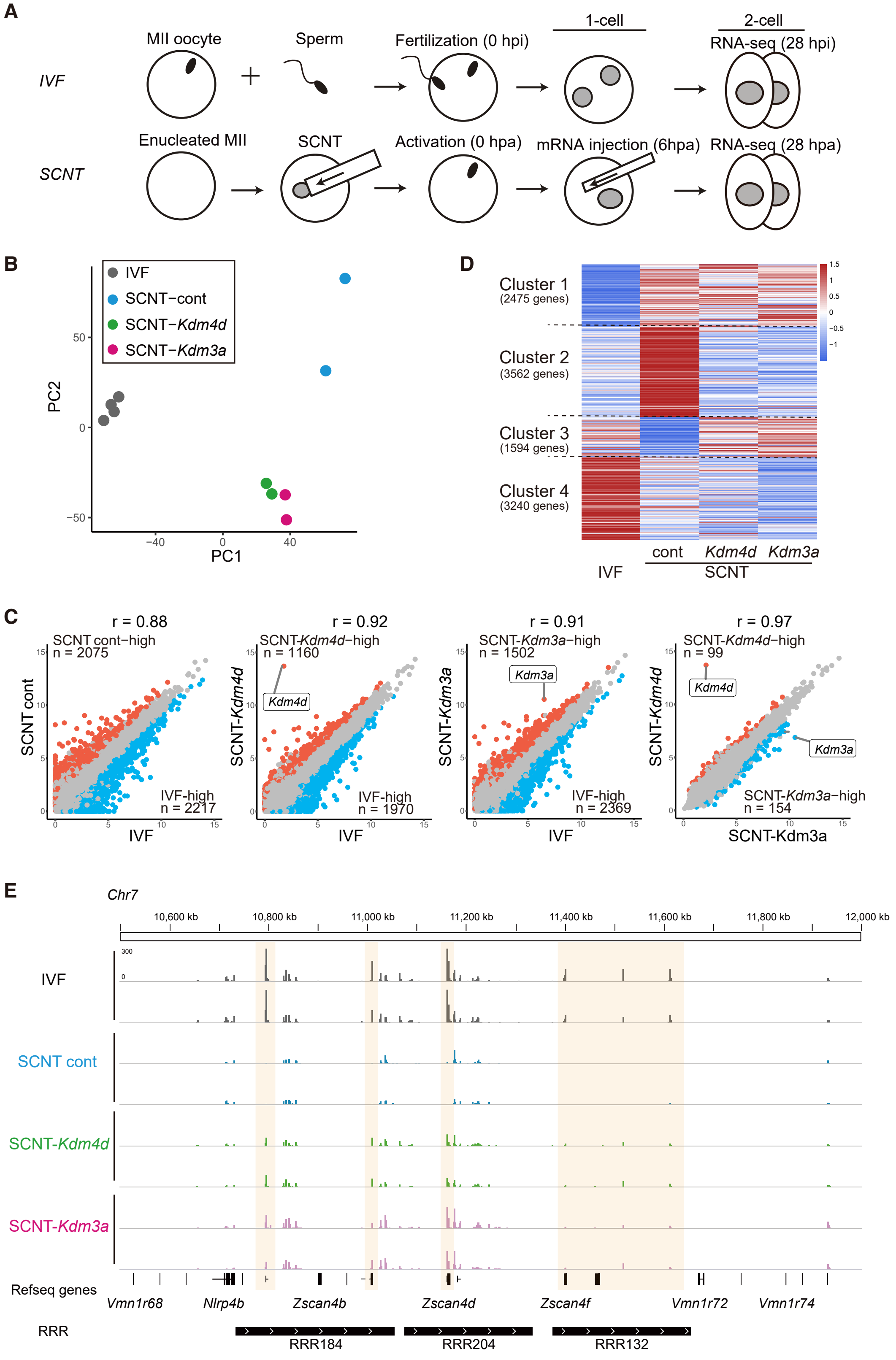

(legend on next page)

(SCNT-*Kdm4d*) at the late two-cell stage (Figure 2A; Table S1). Principal component analysis (PCA) using genes with >1 transcript per kilobase million (TPM) on average among all samples separated control SCNT embryos from IVF embryos (Figure 2B). As expected, SCNT-*Kdm4d* embryos were closer to IVF embryos than control SCNT embryos. Interestingly, the injection of *Kdm3a* induced a significant change in the transcriptome that was similar to that induced by *Kdm4d* (Figure 2B).

Pairwise comparisons revealed that the number of differentially expressed genes (DEGs) between SCNT embryos and IVF embryos was decreased by injection of either *Kdm3a* or *Kdm4d* (3,871 or 3,130, respectively) from control (4,292). Injection of *Kdm3a* or *Kdm4d* also increased the correlation between SCNT transcriptome with IVF embryos (r = 0.91 or 0.92, respectively) compared with the control (r = 0.88) (Figure 2C). Strikingly, SCNT-*Kdm3a* and SCNT-*Kdm4d* embryos had a limited number of DEGs (253 genes) with extremely high correlation coefficients (r = 0.97) (Figure 2C). K-means clustering using the genes expressed in at least one sample (>10 TPM) also indicated that *Kdm3a* and *Kdm4d* induced similar transcriptomic changes. While genes in clusters 1 (2,475 genes) and 4 (3,240 genes) were unchanged by injection of *Kdm3a* or *Kdm4d*, genes in clusters 2 (3,562 genes) and 3 (1,594 genes) were downregulated and upregulated, respectively, in a similar manner by *Kdm3a* or *Kdm4d* injection (Figure 2D). Indeed, genes in the *Zscan4* cluster and *Obox* cluster, which are well-known reprogramming-resistant genes (RRGs) within reprogramming-resistant regions (RRRs) (Matoba et al., 2014), were derepressed similarly by *Kdm3a* and *Kdm4d* in SCNT embryos (Figures 2E and S1A). Quantitative reverse-transcription PCR analysis confirmed the dosage-dependent effect of *Kdm4d* and *Kdm3a* mRNA on ZGA gene derepression (Figures S1B and S1C). Gene Ontology (GO) analysis revealed that downregulated cluster 2 genes are involved in protein catabolism and kinase signaling and that upregulated cluster 3 genes are enriched for the biogenesis of ribonucleoprotein or ribosome (Figure S1D), which suggested that *Kdm* injection accelerates the transition from somatic to embryonic metabolism. These results suggest that H3K9me3 demethylation secondarily induced by the loss of H3K9me1/2 can ameliorate the transcriptional reprogramming in a manner similar to that induced by direct demethylation of H3K9me3.

Kdm3a injection significantly improves the developmental potential of SCNT embryos

Next, we examined the effects of *Kdm3a* or *Kdm4d* injection on the pre-implantation development of SCNT embryos. In control SCNT embryos, most embryos arrested development at the two-cell stage and only about 30% of the two-cell embryos developed to the four-cell stage, and this resulted in a very low blastocyst formation rate of <20% (per the number of two-cell stage embryos). By contrast, a high percentage (>90%) of SCNT embryos injected with *Kdm4d* successfully cleaved to the four-cell stage (Figures 3A and 3B; Table S2), and, as reported previously (Matoba et al., 2014), the blastocyst rate reached over 80% per the number of two-cell stage embryos. Interestingly, similar to the results for *Kdm4d* injection, a high percentage (>90%) of *Kdm3a*-injected SCNT embryos developed to the four-cell stage, and >70% reached the blastocyst stage (Figures 3A and 3B; Table S2). These significant improvements in embryonic development are consistent with the results showing that H3K9me3 is depleted in *Kdm3a*-SCNT embryos and that *Kdm3a* and *Kdm4d* induce similar changes in the SCNT transcriptome. Thus, *Kdm3a*-mediated depletion of H3K9me3 caused by loss of H3K9me1/2 may improve SCNT embryo development to the same level as that observed with *Kdm4d*.

G9ai treatment decreases the H3K9me3 level in mouse SCNT embryos

The results described earlier raised the possibility that removal of the H3K9me3-mediated barrier might be achieved by inhibiting H3K9me1/2 formation in SCNT

Figure 2. *Kdm3a* and *Kdm4d* induced similar transcriptome changes in SCNT embryos

(A) Schematic illustration of the experimental approach. IVF or SCNT embryos were collected at the two-cell stage for RNA-seq analysis (28 hpi or hpa, respectively).

(B) PCA plot of the transcriptome derived from the two-cell stage embryos. Genes with TPM >1 on average among all samples were used. Each dot represents a single sample. Note that the transcriptome of *Kdm3a*-injected SCNT embryos (SCNT-*Kdm3a*) is very similar to that of *Kdm4d*-injected SCNT embryos (SCNT-*Kdm4d*).

(C) Scatterplots comparing the gene expression levels of IVF and SCNT embryos at the two-cell stage. The genes with significant differences (fold change > 2) are presented in red or blue. Note that SCNT-*Kdm3a* and SCNT-*Kdm4d* exhibited highly similar transcriptomes (r = 0.97).

(D) Heatmap comparing the expression levels of all expressed genes (TPM >10 in at least one sample). TPM values were converted to *Z* score. Genes were grouped into four clusters using K-means clustering. Note that clusters 2 and 3 responded similarly to *Kdm3a* and *Kdm4d* in the SCNT embryos.

(E) A genome browser view of RNA-seq data at the *Zscan4* cluster on chromosome 7 containing *Kdm3a*/*4d*-responsive genes (yellow box). See Figure S1 and Table S1.

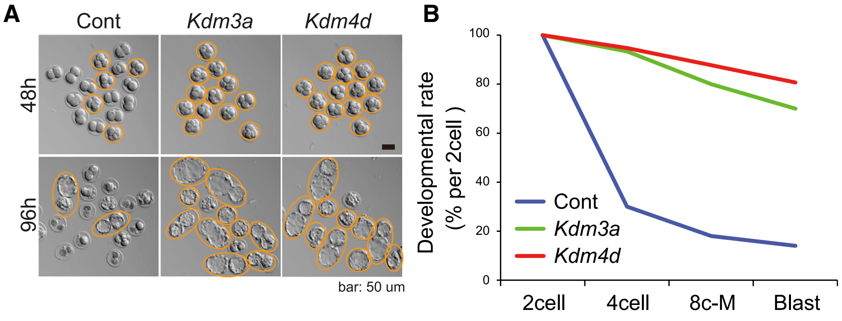

Figure 3. *Kdm3a* markedly improved the pre-implantation development of SCNT embryos derived from cumulus cells

(A) Representative images of SCNT embryos at 48 and 96 h of *in vitro* culture. Normally developing embryos are marked with orange circles. Scale bar, 50 μm.

(B) Line plot showing the percentages of embryos that reach the indicated stages. 2cell, two-cell stage at 24 hpa; 4cell, 4-cell stage at 48 hpa; 8c-M, 8-cell stage or morula stage at 72 hpa; Blast, blastocyst stage at 96 hpa. See Table S2.

embryos. In mammals, H3K9me1/2 is formed mainly by the cooperation of two histone methyltransferases, G9a/EHMT2 and GLP/EHMT1 (Tachibana et al., 2005). Recently, in addition to the available G9a/GLP inhibitors such as A366 (Sweis et al., 2014) (half maximal inhibitory concentration [IC50]: 3.3 nM) or UNC0638 (Vedadi et al., 2011) (IC50: <15 nM), a series of highly selective and potent G9a inhibitors (G9ai) (RK series; e.g., IC50 of RK-701: 23–27 nM) have been developed (Nishigaya et al., 2023; Takase et al., 2023). We used these selective G9ai to reduce H3K9 methylation levels in SCNT embryos.

First, we evaluated the effect of various G9ai on embryonic development and H3K9 methylation levels in IVF embryos (Figure S2). None of the G9ai affected development to the two-cell stage. However, whereas most inhibitors did not affect development to the blastocyst stage, UNC0638 completely blocked blastocyst formation (Figures S2A and S2B). To examine the effect of G9ai treatment on post-implantation development, we transferred the G9ai-treated IVF embryos (other than UNC0638) to pseudopregnant females. We found that G9ai-treated embryos developed to term at a similar efficiency to control (Figure S2C; Table S3) without any phenotypic abnormality including body and placenta weight (Table S3). We also examined the H3K9me1/2/3 levels in IVF embryos at the one-cell, two-cell, and blastocyst stages. G9ai treatment did not change the levels of H3K9me1/2/3 at the one-cell stage (Figures S2D–S2F) and only slightly reduced H3K9me2/3 levels at the two-cell stage (Figures S3A–S3C). H3K9me1/2 staining of the blastocysts showed that H3K9me1/2 levels were significantly reduced by almost all G9ai, but no such effect was observed for RK-0133114, which is the inactive *R*-enantiomer form of RK-701 (Nishigaya et al., 2023; Takase et al., 2023) (Figures S3D–S3F). H3K9me3 level was only slightly affected by these G9ai in this setting (Figures S3E and S3F).

Next, we examined the effect of G9ai on H3K9me1/2/3 levels in SCNT embryos. First, we used immunostaining to assess the H3K9me1/2 levels at the one-cell stage, 8 h after the initiation of an inhibitor treatment (Figure 4A). The H3K9me1 level was slightly but not significantly decreased by RK-701 treatment at this stage, but the levels of H3K9me2 and H3K9me3 decreased significantly by 39% and 29%, respectively (Figure 4B). Such decreases in H3K9me1/2/3 levels by RK-701 became even more evident at the two-cell stage (Figure 4C). Consistent with the decrease of H3K9 methylation, several histone demethylases were detected at these stages (Figures S4A and S4B).

It has been shown that treatment with HDAC inhibitors, such as trichostatin A (TSA), significantly increases the mouse cloning rate (Kishigami et al., 2006; Rybouchkin et al., 2006) Interestingly, the effects of G9ai on H3K9 methylation were enhanced greatly when combined with TSA. TSA treatment significantly increased the H3K9 acetylation levels at the one-cell stage, but not at the two-cell stage (Figures S4C–S4F). Dual treatment with G9ai and TSA markedly reduced H3K9me1, H3K9me2, and H3K9me3 levels by 43%, 72%, and 58%, respectively, at the two-cell stage (Figures 4C and 4D). It is noteworthy that H3K9me1 was elevated by TSA treatment in one-cell stage embryos. Comparison of the signal intensities between H3K9me3 and DAPI within the two-cell nucleus confirmed that the combined treatment of TSA and G9ai globally reduced the H3K9me3 level, although some peri-nucleolar regions maintained faint H3K9me3 signals (Figure 4E). These results suggest that G9ai inhibits the formation of H3K9 methylation and has a synergistic effect with TSA on H3K9 demethylation.

G9ai derepress ZGA genes in SCNT embryos

To understand the transcriptional effect of G9ai, we performed RNA-seq in two-cell stage embryos. To gain better insight into the effect of G9a inhibition, we used three different G9ai including RK-701, A366, and UNC0638 (Table S1). Pairwise comparisons of TSA and/or G9ai-treated SCNT embryos with IVF embryos revealed that, unexpectedly, each treatment reduced the number of DEGs against IVF embryos only slightly (Figure S5A). Therefore, instead of analyzing the whole transcriptome, we focused on RRGs

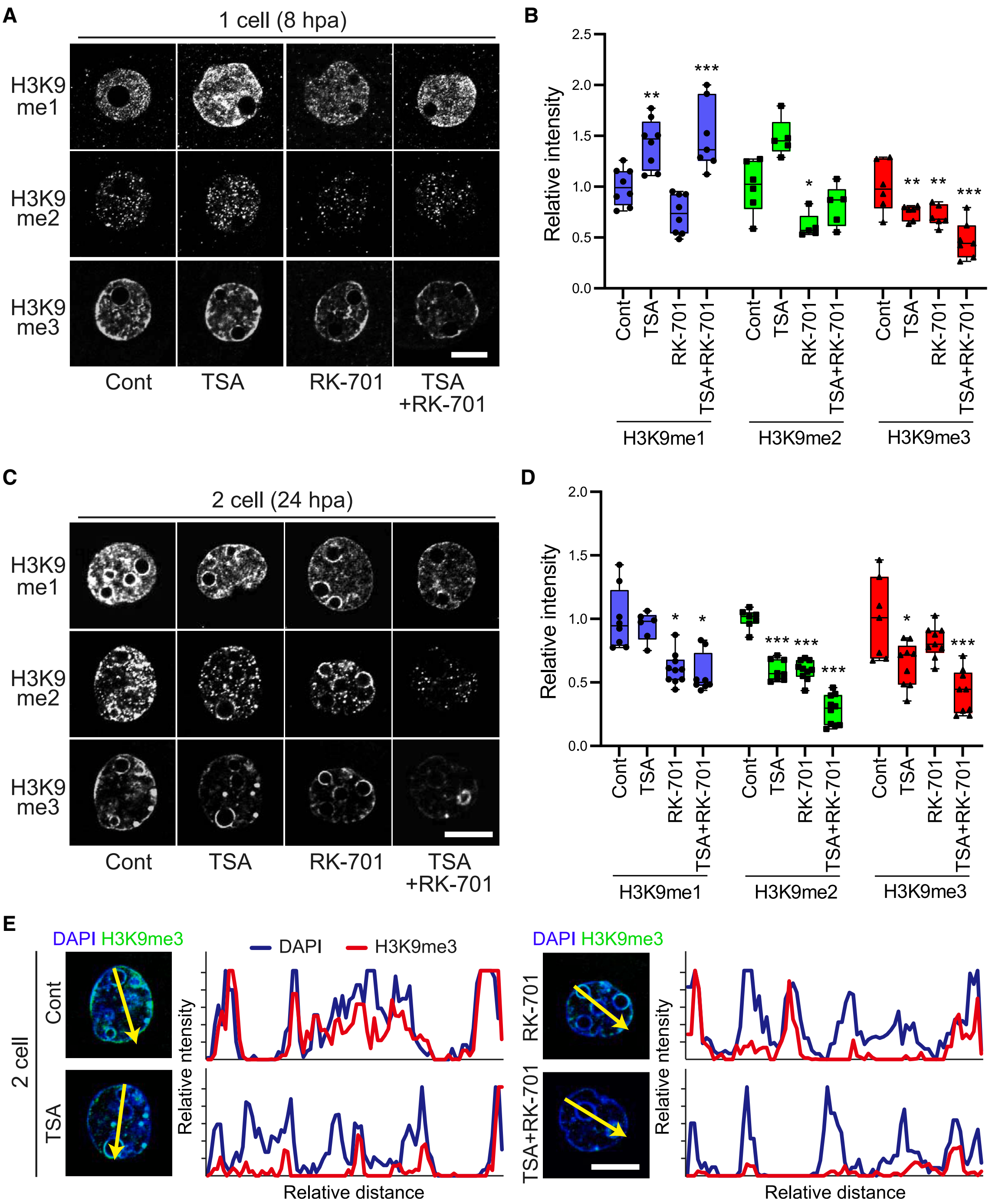

Figure 4. G9ai significantly reduced H3K9me3 levels in SCNT embryos at the two-cell stage

(A) Representative images of nuclei of SCNT embryos immuno-stained using anti-H3K9me1, anti-H3K9me2, and anti-H3K9me3 antibodies at the one-cell stage. Scale bar, 5 μm.

(B) Boxplots showing the relative intensities of H3K9me1/2/3 levels in each sample at the one-cell stage. ***p* < 0.01, ****p* < 0.001 compared to control.

(C) Representative images of the nucleus of SCNT embryos immuno-stained using anti-H3K9me1, anti-H3K9me2, and anti-H3K9me3 antibodies at the two-cell stage. Scale bar, 5 μm.

(legend continued on next page)

(879 genes; 2C/1C $\geq$ 2, $p < 0.05$, in Matoba et al. (2014) and SCNT/IVF $\geq$0.5, $p < 0.05$ in this study; Table S4). PCA analysis using RRGs separated IVF and control SCNT embryos (Figure 5A). TSA alone or G9ai alone, either RK-701 or A366, induced a slight transition in the transcriptome of SCNT embryos. Interestingly, G9ai and TSA co-treatment synergistically transformed the SCNT transcriptome. A similar effect was observed for all G9ai examined, including RK-701+TSA, A366+TSA, and UNC0638+TSA (Figure 5A). PCA using the whole transcriptome (not RRGs) together with those of *Kdm3a*- and *Kdm4d*-injected embryos (genes expressed at TPM >1 on average) showed that G9ai+TSA samples were closely positioned with *Kdm3a*/*4d*-injected SCNT embryos (Figure S5B). This finding suggests that G9ai+TSA treatment restored the transcriptome via the same pathway as with *Kdm3a* or *Kdm4d*.

K-means clustering of RRGs separated these into seven clusters. Six G9ai- or/and TSA-responsive clusters are shown in Figure 5B. Cluster 4 (89 genes) and cluster 5 (84 genes) were derepressed by TSA and G9ai, respectively, but cluster 6 (38 genes) responded similarly to these two treatments. Interestingly, cluster 2 (246 genes) was activated only when TSA and G9ai were treated simultaneously. GO analysis revealed that cluster 2 was enriched for "blastocyst formation" genes (Figure S5C), which suggests that the dual treatment activated the developmentally important genes in the SCNT embryos. Indeed, ZGA-related genes such as *Zscan4a/d* and *Obox3* were significantly derepressed by G9ai (fold change >2, adjusted $p < 0.05$; Figures 5C, 5D, and S5D), and TSA+G9ai treatment further activated other ZGA genes including *Obox6*, *Obox8*, and *Eif1a* (Figures 5E and 5F). Interestingly, several germline genes (*Cox7b2*, *Terb2*, *Cstf2t*, etc.) resistant to *Kdm4d* (Akter et al., 2021; Matoba et al., 2018) were derepressed by this dual treatment (Figure S5E). These results suggest that G9ai allows the efficient activation of ZGA genes in SCNT embryos, especially when combined with TSA.

G9ai and TSA synergistically improve mouse cloning efficiency. Having demonstrated that G9ai treatment can significantly reduce H3K9me3 and normalize the transcriptome to the one similar to that of *Kdm4d/3a*-SCNT, we next asked whether such inhibitor treatment can improve the development of SCNT embryos. First, we optimized the condition of G9ai treatment using cumulus clone (CC) embryos (Figures 6A, S6A, and S6B). In the control, only 36% of two-cell stage CC embryos successfully developed to the four-cell stage (Figures 6A and S6A). RK-701 treatment for 6 and 24 h increased the four-cell rate to 60% and 80%, respectively (Figure S6A). Strikingly, simultaneous treatment of G9ai and TSA increased the four-cell rate further up to 95% (Figures 6A and S6A). When these embryos were cultured until 96 hpa, the RK-701 treatment during the initial 24 h improved the blastocyst rate from 12% to >50% (Figures 6A and S6B). Interestingly, RK-701 treatment for >24 h did not improve, but instead decreased the developmental rate (Figure S6B), which indicated that 24 h of G9ai treatment was optimal.

Intriguingly, the blastocyst rate increased to about 80% when the embryos were co-treated with TSA and RK-701 (Figures 6A, 6B, and S6B; Table S2). Such a synergistic improvement by RK-701 and TSA was observed in SCNT embryos derived from cumulus cells as well as Sertoli cells and MEFs, all of which exhibited blastocyst rates >80% (Figure 6B). We also tested other available G9ai and found that A366 as well as UNC638, which was toxic for the long-term treatment of IVF embryos (Figure S2B) and single treatment in SCNT (Figure S6C), showed the same level of improvement as RK-701 when combined with TSA (Figure 6B). Thus, the optimized treatment with G9ai and TSA greatly increased the developmental rate of SCNT embryos.

Next, to examine the effects of the optimized condition on the post-implantation development of SCNT embryos, we performed embryo transfer at the two-cell stage using cumulus cells as the nuclear donor. In the control SCNT embryos, 35% of embryos transferred to the surrogate mother implanted (Figure S6D), and only 0.7% resulted in birth (Figures 6C and 6D; Table S5). These implantation and birth rates were significantly lower than that in IVF control (Figure S2; Table S3). Treatment with RK-701 alone increased the implantation and birth rates of the SCNT embryos to 49.4% and 3.9%, respectively (Figures S6D and 6D; Table S5). The combination of RK-701 with TSA increased the implantation rate further to 55.8% and the birth rate to 14.5% (Figures S6D, 6C, and 6D; Table S5). Interestingly, other G9ai including A366 and UNC0638 also improved the birth rate >10% when combined with TSA (Figure 6D). Such a significant improvement in the birth rate was similarly observed when Sertoli and MEF cells were used as donors (from 2.0% to 14.1% and from 0% to 7.1%, respectively; Figure 6D; Table S5). The SCNT embryos generated through G9ai+TSA exhibited a large placental phenotype (Figures S6E and S6F; Table S5). The cloned pups normally grew into fertile adults (Figure 6E; Table S5). These results

(D) Boxplots showing the relative intensities of H3K9me1/2/3 levels in each sample at the one-cell stage. *$p < 0.05$, ***$p < 0.001$ compared with the control.
(E) Line profiles (yellow arrows) of DAPI and H3K9me3 channels showing the relative intensities in the nucleus of the two-cell stage embryos. Scale bar, 5 μm. See Figures S2–S4, Table S3.

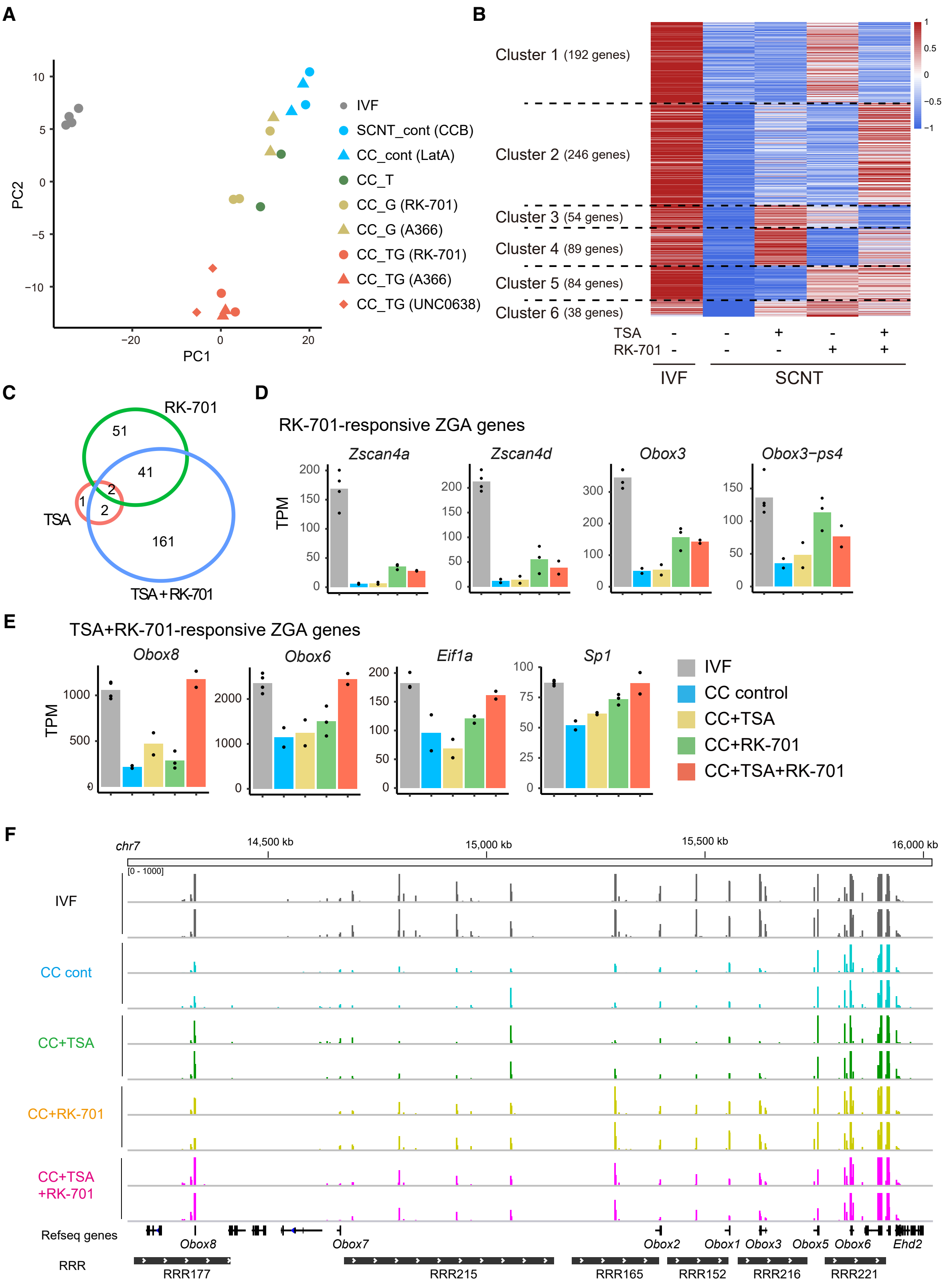

(legend on next page)

suggest that the optimized treatment with G9ai and TSA greatly improves the efficiency of SCNT-mediated mouse cloning from any type of cells.

DISCUSSION

In this study, we explored the possible mechanisms to decrease the H3K9me3 level in SCNT embryos and found that demethylation of H3K9me1/2 by *Kdm3a* results in a secondary decrease of H3K9me3 that leads to a significant improvement in genome-wide transcription and embryonic development of SCNT embryos. These results suggested that inhibition of the formation of H3K9me1/2 in the SCNT embryos may help to reduce H3K9me3, which would improve cloning efficiency. This concept is consistent with our findings showing that the optimized treatment to inhibit G9a, which is responsible for the formation of H3K9me1/2 in mammalian cells (Tachibana et al., 2005), decreased not only H3K9me1/2 but also H3K9me3 in the SCNT embryos at the two-cell stage. Such reduction of H3K9me3 might be SCNT embryo-specific as *G9a/Ehmt2* knockout did not change the H3K9me3 level in mouse embryonic stem cells (Tachibana et al., 2005). Indeed, G9ai treatment only slightly affected H3K9me3 levels in the IVF embryos at the two-cell stage (Figures S3A–S3C). The effect of G9ai was synergistic with that of HDACi TSA because simultaneous treatment with the two inhibitors markedly decreased the H3K9me3 level and significantly improved ZGA gene expression and developmental efficiency of the SCNT embryos from cumulus cells, whose rate reached 14%. Given its simple, easy, and high-throughput nature, this G9ai+TSA method has the potential to become a fundamental technology for future cloning research and applications.

We have shown that H3K9me1/2 demethylation by *Kdm3a* at the one-cell stage induced a secondary loss of H3K9me3 at the two-cell stage. The loss of a suitable substrate for H3K9me3 generation should have compromised the new deposition of H3K9me3 and significantly diluted H3K9me3 after replication. However, it is unclear why the level of heterochromatic H3K9me3 already present in the donor cells was not maintained but was almost completely depleted. During DNA replication, parental histones are segregated equally onto both daughter strands, while new histones are deposited in between these parental histones (Escobar et al., 2021; Shan et al., 2023). SUV39H1 binds to H3K9me3 and initiates H3K9 methylation in the surrounding unmethylated histones. This H3K9me3-spreading mechanism requires a specific density of H3K9me3 to maintain the heterochromatic state (Cutter DiPiazza et al., 2021). We speculate that loss of H3K9me1/2 may have compromised this spreading mechanism and that endogenous histone demethylases present in the ooplasm, such as KDM1A, KDM4A (Sankar et al., 2020), and KDM7A/B/C (Figure S4A), removed the parental H3K9me3 in the absence of H3K9me3-spreading activities. Consistently, we found that the faint signals of H3K9me3 remained in the *Kdm3a*-injected SCNT embryos at the two-cell stage and were located in the nucleolus-surrounding regions where H3K9me3 is densely enriched in the control SCNT embryo. Further experiments are required to examine this model. Although the transcriptional and developmental consequences of *Kdm3a*- and *Kdm4d*-injection on SCNT embryos were likely through H3K9me3 demethylation based on the clear overlap between RRRs and H3K9me3 (Matoba et al., 2014), we cannot exclude the pathway through H3K9me2 demethylation that might at least partially contribute to these effects.

Treatment of SCNT embryos with G9ai significantly reduced H3K9me3 levels, which helped activation of ZGA genes and improved mouse cloning rate. These effects were boosted markedly by co-treatment with an HDACi, TSA, which has been shown to improve the cloning rate (Kishigami et al., 2006; Rybouchkin et al., 2006). It is unclear how such synergistic effects were achieved. As expected from the activity of G9a, G9ai treatment significantly reduced H3K9me1/2 levels in the SCNT embryos. Interestingly, G9ai alone significantly reduced the H3K9me3 level. Based on the extremely high selectivity of G9ai to G9a

Figure 5. G9ai and TSA synergistically improved the transcriptome of SCNT embryos at the two-cell stage

(A) PCA plot of the transcriptome derived from the two-cell stage embryos. RRGs that were upregulated from one-cell to two-cell stage IVF embryos (2C/1C $\geq$ 2, $p < 0.05$ in Matoba et al. (Matoba et al., 2014) dataset, GEO: GSE59073) and downregulated in SCNT embryos compared with IVF embryos at the two-cell stage (SCNT/IVF $\leq$0.5, $p < 0.05$ in the dataset generated in this study) were used. Each dot represents a single sample. Note that G9ai and TSA synergistically converted the transcriptome of SCNT embryos and that three different G9ai-treated samples co-treated with TSA (CC_TG [RK-701], CC_TG [A366], and CC_TG [UNC0638]) were closely clustered together.

(B) Heatmap comparing the expression levels of RRGs. TPM values were converted to *Z* score. RRGs were grouped into seven clusters using K-means clustering. Six clusters that responded to either TSA or G9ai are shown. Note that cluster 2 was upregulated only by TSA+RK-701.

(C) Venn diagram showing the overlap between the RRGs that were derepressed by TSA, RK-701, or TSA+RK-701 compared with the SCNT control.

(D) Bar graphs showing the expression levels of RK-701-responsive (activated by both RK-701 and TSA+RK-701) ZGA genes.

(E) Bar graphs showing the expression levels of TSA+RK-701-responsive (activated only by TSA+RK-701) ZGA genes.

(F) A genome browser view of RNA-seq data at the *Obox* cluster on chromosome 9. See Figure S5, and Tables S1 and S4.

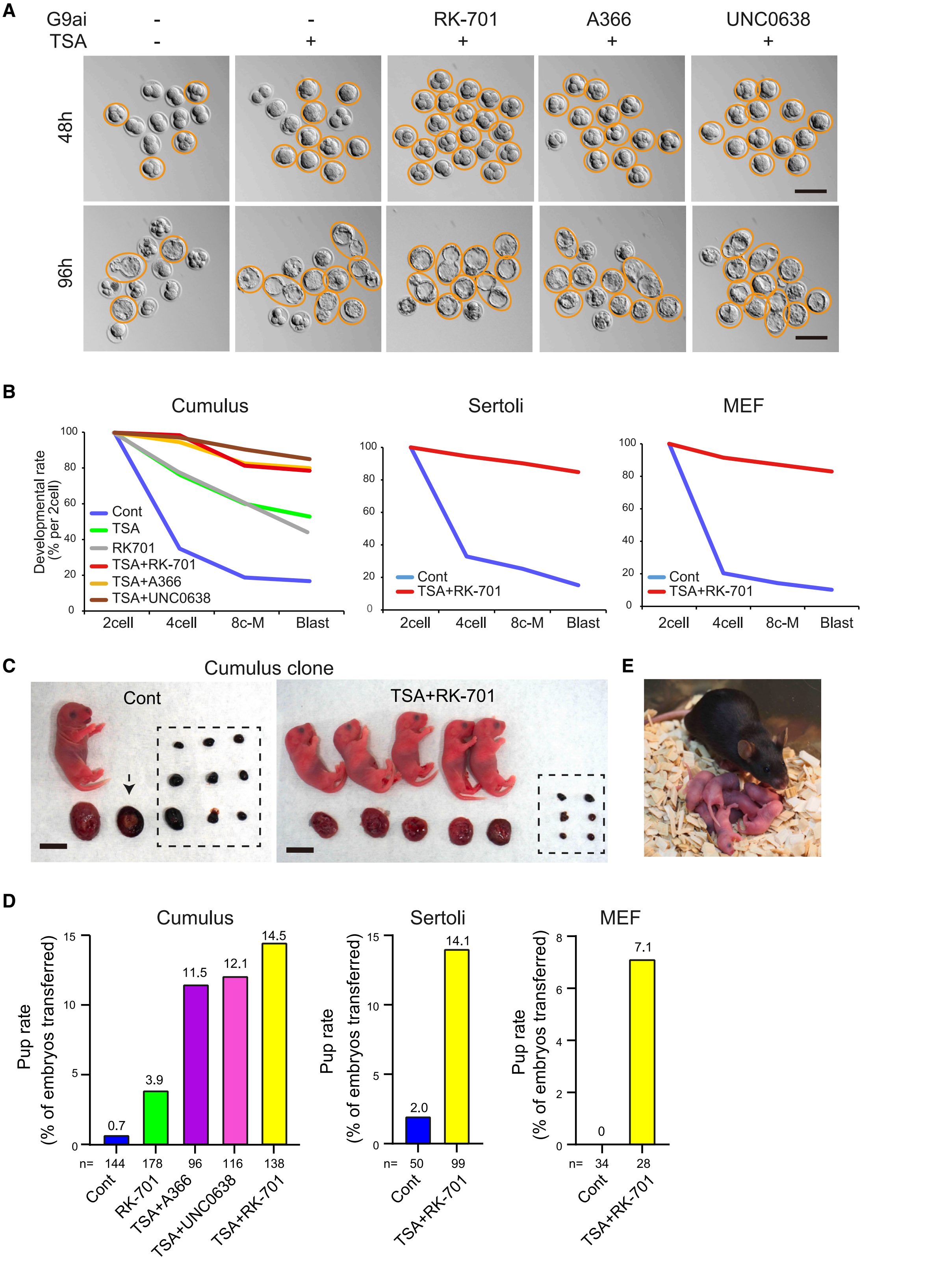

(legend on next page)

(Nishigaya et al., 2023; Takase et al., 2023), it is unlikely that G9ai directly inhibited SUV39H1/2 or SETDB1. Since G9a is known to form a multimeric complex with other histone methyltransferases including SUV39H1/2 (Fritsch et al., 2010), G9ai may have partially inhibited the catalytic activities of the other components within such complexes. By contrast, treatment with TSA alone significantly decreased the H3K9me2/3 levels (Figure 4D) while increasing the H3K9ac levels (Figures S4C–S4F). Deacetylation of histone lysine residues is a prerequisite for the installation of methylation in the target histones (Bannister and Kouzarides, 2011; Rice and Allis, 2001), and TSA may inhibit H3K9me2/3 formation indirectly via blocking the deacetylation of H3K9ac. These two independent mechanisms likely boosted the demethylation of H3K9me3 and active transcription of developmentally critical ZGA genes such as those in the *Obox* and *Zscan4* families (Figure 5).

Small-molecule inhibitors of H3K9 methyltransferases have been examined in attempts to improve SCNT cloning efficiency. Chaetocin is the first lysine-specific histone methyltransferase inhibitor that can inhibit multiple targets including SUV39H1/2 and G9a (Cherblanc et al., 2013; Greiner et al., 2005; Iwasa et al., 2010). Although chaetocin treatment has been reported to improve slightly but significantly the pre-implantation development of SCNT embryos in pigs (Jeong et al., 2020), it has an adverse effect in sheep (Zhang et al., 2018), bovine (Jafarpour et al., 2020), and mouse SCNT models (unpublished data of AO), possibly because of its low selectivity. BIX-01294 was the first G9a-specific inhibitor identified in 2007 (Kubicek et al., 2007). Similar to chaetocin, BIX-01294 also has a detrimental effect on embryonic development in mice (Huang et al., 2017), although it has been reported to improve pig cloning to some extent (Cao et al., 2017; Huang et al., 2016). These adverse effects likely relate to its low selectivity and narrow range between the efficacy (IC50 to G9a: 1.7 μM) and toxicity (toxic at 4.1 μM) doses (Cao et al., 2019). In contrast to these previous reports, we observed a significant improvement in SCNT efficiency by RK-701, possibly because of its high selectivity and low toxicity (Nishigaya et al., 2023; Takase et al., 2023). Similar improvement to a birth rate >10% was achieved with another two G9ai, A366 and UNC0638. Interestingly, although UNC0638 was toxic when used alone with IVF or SCNT embryos (Figures S2 and S6C), it had an ameliorating effect when co-treated with TSA (Figure 6). This ability of a specific combination of drugs to increase the therapeutic efficacy and reduce toxicity compared with a single drug is known as a synergistic drug combination (Lehár et al., 2009). Therefore, some inhibitors that had failed to improve SCNT because of their toxicity (such as chaetocin) might be effective in SCNT if an appropriate combination with other drugs is identified.

In summary, we have provided insights into the molecular mechanisms that may be useful for attenuating H3K9 methylation in SCNT embryos, and we have established an optimized noninvasive method to significantly improve mouse cloning efficiency using the combination of G9ai and TSA. This method has several advantages to the *Kdm4*-injection approach: it has better technical feasibility, is less time-consuming, and causes less damage to embryos. The high cloning efficiency reaching >14% in cumulus clones without any genetic modulation proves the reliability of this approach. The method described here is technically easy and can be readily applied to various mouse strains and other animal species, and this method may provide a basis for future cloning studies.

EXPERIMENTAL PROCEDURES

Resource availability

Lead contact

Further information and requests for resources and reagents should be directed to and will be fulfilled by the lead contact, Shogo Matoba (shogo.matoba@riken.jp).

Materials availability

The materials included in this study are available from the corresponding author upon reasonable request.

Data and code availability

The accession number for the RNA-seq datasets reported in this paper is GEO: GSE248499. No new code was generated in this study.

Figure 6. G9ai and TSA co-treatment markedly improved the embryonic development of SCNT embryos derived from cumulus cells, Sertoli cells, and MEFs

(A) Representative images of SCNT embryos derived from cumulus cells at 48 and 96 h of *in vitro* culture. Normally developing embryos are marked with orange circles. Scale bar, 100 μm.

(B) Line plots showing the percentages of embryos that reached the indicated stages. Note that any G9ai examined significantly improved the blastocyst rate when combined with TSA. 2cell, two-cell stage at 24 hpa; 4cell, 4-cell stage at 48 hpa; 8c-M, 8-cell stage or morula stage at 72 hpa; Blast, blastocyst stage at 96 hpa.

(C) Representative images of a single litter of SCNT embryos derived from cumulus cells at birth. Left panel; control litter, right panel; TSA+RK-701-treated litter. Those in the dashed box represent embryos absorbed after implantation.

(D) Bar graphs showing the birth rate of SCNT embryos examined by the caesarean section on E19.5.

(E) An image of an adult female mouse derived by SCNT from a cumulus cell through TSA+RK-701 treatment and its pups generated through natural mating with a wild-type male. See Figure S6, and Tables S2 and S5.

MICE

All animal experiments were approved by the Institutional Animal Care and Use Committee of RIKEN Tsukuba Institute. ICR, B6D2F1 (BDF1), DBA/2, and C57BL/6N (B6N) mice were purchased from Japan SLC Inc. Mice were housed in specific pathogen-free conditions with controlled lighting (daily light from 07:00 to 21:00).

SCNT

Mouse SCNT was performed as previously described (Matoba et al., 2018).· In brief, recipient MII oocytes were collected from adult BDF1 females after superovulation. Isolated MII oocytes were enucleated in HEPES-buffered KSOM containing 7.5 mg/mL of cytochalasin B (Calbiochem #250233). The nuclei of cumulus or Sertoli cells were injected into the enucleated oocytes using a Piezo-driven micromanipulator. MEFs were fused with enucleated oocytes using an inactivated Sendai virus envelope (GenomOne CF; Ishihara Sangyo #CF001). After incubation for 1 h in KSOM, reconstructed SCNT oocytes were activated by incubating in Ca-free KSOM containing 3 mM strontium chloride ($SrCl_2$) and 5 mg/mL cytochalasin B (CCB) for 1 h, and cultured further in KSOM with 5 mg/mL cytochalasin B for 4 h. In some experiments, latrunculin A was added to KSOM instead of CCB, and these SCNT embryos were treated for 8 h in total after the initiation of $SrCl_2$ activation. Some SCNT embryos were injected with about 10 pL (similar to the volume of a pronucleus) of 1,500 ng/mL mouse *Kdm4d* or *Kdm3a* mRNA at 5–6 hpa.

Inhibitor treatment

The G9ai used in this study are listed in Table S6. The original stock solutions of G9ai were prepared at 10 mM in dimethyl sulfoxide (DMSO) and kept at −80°C. Each G9ai was further diluted to 500 μM with DMSO to make diluted stock solution aliquots that were kept at −80°C until use. The diluted stock solution of G9ai (500 μM) was added to each medium at 1/500 dilution to make the final concentration at 1 μM. For the initial examination of the effect of G9ai in IVF embryos, fertilized zygotes were treated with 1 μM G9ai from 5 hpi until 24 hpi (for embryo transfer experiment) or until the end of *in vitro* culture up to 96 hpi. For the SCNT experiments, the duration of the G9ai treatment was optimized to 24 h from the initiation of activation. G9ai were added to the $SrCl_2$-containing activation medium following KSOM at 1 μM for up to 24 hpi. In some experiments, 25 nM TSA was added to the medium for the first 8 h.

Preparation of *Kdm4d* and *Kdm3a* mRNA

mRNA was synthesized by *in vitro* transcription (IVT), as described previously (Matoba et al., 2014). Briefly, pcDNA plasmid containing full-length mouse *Kdm4d* or *Kdm3a* followed by a polyA tail was linearized by *Xba*I or *Xho*I, respectively. After purification, the linearized plasmid DNA was used as a template for IVT using mMESSAGE mMACHINE T7 Ultra Kit (Thermo Fisher Scientific #AM1345). The synthesized mRNA was dissolved in nuclease-free water and quantified using a NanoDrop ND-1000 spectrophotometer (NanoDrop Technologies). The purified mRNA was diluted to 1,500 ng/mL and 1 μL aliquots were stored at −80°C until use.

Immunostaining

Embryos were fixed with 4% paraformaldehyde in phosphate-buffered saline (PBS) for 20 min at room temperature. The fixed embryos were permeabilized for 15 min by incubation with 0.5% Triton X-100. After blocking in PBS/BSA for 1 h at room temperature, these were incubated in a mixture of primary antibodies at 4°C overnight. The primary antibodies used are as follows: rabbit anti-H3K9me1 (Abcam, ab9045, 1:500), mouse anti-H3K9me2 (Abcam, ab1220, 1:500), rabbit anti-H3K9me3 (Abcam, ab8898, 1:500), and mouse anti-H3K9ac (Active Motif, 61952, 1:500). Following three washes, the samples were incubated with secondary antibodies that include donkey anti-rabbit Alexa 488 (Thermo Fisher Scientific, A-21206) and donkey anti-mouse Alexa 555 (Thermo Fisher Scientific, A-31570) for 1 h at room temperature. The nuclei were co-stained with DAPI (Vector Laboratories). The fluorescent signals were observed with Nikon C2 confocal microscopy and quantified with NIS-Elements AR (Nikon) and ImageJ software. Statistical significance was evaluated with unpaired Student's t test.

RNA-seq library preparation

Two-cell stage embryos derived from IVF and SCNT were collected at 28 hpi or hpa, washed twice in 0.05% BSA in PBS, and flash-frozen in liquid nitrogen. Five or 10 embryos were mixed as a single sample. After thawing, polyadenylated RNAs were reverse transcribed and amplified, using SMART-Seq HT kits (R400748; Takara Bio Inc.). The quality of sequence libraries was examined using a 2100 Bioanalyzer with High-Sensitivity DNA kits (5067-4626; Agilent Technologies). Paired-end 150 bp sequencing was performed on a HiSeq X platform (Illumina).

RNA-seq analysis

Adapter sequences and low-quality reads were removed using Trimmomatic (version 0.36) (Bolger et al., 2014). The resulting sequence reads were aligned uniquely to the mm10 mouse genome using STAR aligner software (version 2.7.5c) (Dobin et al., 2013) with the following parameters: "–alignIntronMin 20 –alignIntronMax 1000000 – alignMatesGapMax 1000000 –alignSJoverhangMin 8 –alignSJDBoverhangMin 1 – twopassMode Basic –readFilesCommand zcat –outFilterType BySJout – outFilterMultimapNmax 1 –outFilterMismatchNmax 999 – outFilterMismatchNoverReadLmax 0 –outSAMtype BAM Unsorted –winAnchorMultimapNmax 50." The mapped reads were counted using featureCounts (Liao et al., 2014). The expression levels of genes were calculated as normalized TPM data. DEGs were identified using the DESeq2 package (version 1.16.1) (Love et al., 2014). Expressed transcripts with at least one read on average were used for analysis. Coverage tracks normalized by DEseq2 were generated with BAMscale. GO analyses were performed using clusterProfiler (version 4.8.3) in the data presented in Figure S1D, and Metascape (version 3.5.20230501) in the data presented in Figure S5C. Sex chromosomes were excluded from this analysis because IVF embryos include male and female cells, whereas all SCNT embryos are female, and the ratio of X and Y chromosomes differs between IVF and SCNT embryos. The gene expression levels in all samples analyzed in this study are shown in Table S1.

SUPPLEMENTAL INFORMATION

Supplemental information can be found online at https://doi.org/10.1016/j.stemcr.2024.04.003.

ACKNOWLEDGMENTS

We thank Dr. Gen Watanabe for providing the anti-inhibin serum, Drs. Yoichi Shinkai, Makoto Tachibana, and Ryo Maeda for helpful discussions, and Toshiko Tomishima, Yukiko Dodo, and Ayano Tsukahara for technical assistance. This study was supported by the Japan Agency for Medical Research and Development (AMED) under grant number JP21dm0207118 (S.M.), Japan Science and Technology Agency (JST) under Grant Number JPMJFR221G (S.M.), and KAKENHI grant numbers JP20H03159 (S.M.) and JP19H05758 (A.O.).

AUTHOR CONTRIBUTIONS

S.M. and A.O. conceived the project, designed the experiments, and wrote the manuscript. S.M. performed most of the experiments. D.S. prepared the RNA-seq library and analyzed the sequencing data. T.T. helped in the sequencing data analysis. M.H., N.W., and A.H. helped in the SCNT experiments. F.S., A.N., A.I., and M.Y. provided G9ai.

DECLARATION OF INTERESTS

F.S., A.I., and M.Y. are inventors on a PCT international patent application (WO/2021/106988) that covers RK-701, a compound studied in this paper.

Received: December 1, 2023
Revised: March 28, 2024
Accepted: April 4, 2024
Published: May 9, 2024

REFERENCES

Akter, M.S., Hada, M., Shikata, D., Watanabe, G., Ogura, A., and Matoba, S. (2021). CRISPR/Cas9-based genetic screen of SCNT-reprogramming resistant genes identifies critical genes for male germ cell development in mice. Sci. Rep. *11*, 15438. https://doi.org/10.1038/s41598-021-94851-9.

Bannister, A.J., and Kouzarides, T. (2011). Regulation of chromatin by histone modifications. Cell Res. *21*, 381–395. https://doi.org/10.1038/cr.2011.22.

Bolger, A.M., Lohse, M., and Usadel, B. (2014). Trimmomatic: a flexible trimmer for Illumina sequence data. Bioinformatics *30*, 2114–2120. https://doi.org/10.1093/BIOINFORMATICS/BTU170.

Bulut-Karslioglu, A., De La Rosa-Velázquez, I.A., Ramirez, F., Barenboim, M., Onishi-Seebacher, M., Arand, J., Galán, C., Winter, G.E., Engist, B., Gerle, B., et al. (2014). Suv39h-dependent H3K9me3 marks intact retrotransposons and silences LINE elements in mouse embryonic stem cells. Mol. Cell *55*, 277–290. https://doi.org/10.1016/j.molcel.2014.05.029.

Cao, H., Li, L., Yang, D., Zeng, L., Yewei, X., Yu, B., Liao, G., and Chen, J. (2019). Recent progress in histone methyltransferase (G9a) inhibitors as anticancer agents. Eur. J. Med. Chem. *179*, 537–546. https://doi.org/10.1016/j.ejmech.2019.06.072.

Cao, Z., Hong, R., Ding, B., Zuo, X., Li, H., Ding, J., Li, Y., Huang, W., and Zhang, Y. (2017). TSA and BIX-01294 induced normal DNA and histone methylation and increased protein expression in porcine somatic cell nuclear transfer embryos. PLoS One *12*, e0169092. https://doi.org/10.1371/journal.pone.0169092.

Cherblanc, F.L., Chapman, K.L., Brown, R., and Fuchter, M.J. (2013). Chaetocin is a nonspecific inhibitor of histone lysine methyltransferases. Nat. Chem. Biol. *9*, 136–137. https://doi.org/10.1038/nchembio.1187.

Chung, Y.G., Matoba, S., Liu, Y., Eum, J.H., Lu, F., Jiang, W., Lee, J.E., Sepilian, V., Cha, K.Y., Lee, D.R., and Zhang, Y. (2015). Histone demethylase expression enhances human somatic cell nuclear transfer efficiency and promotes derivation of pluripotent stem cells. Cell Stem Cell *17*, 758–766. https://doi.org/10.1016/j.stem.2015.10.001.

Cutter DiPiazza, A.R., Taneja, N., Dhakshnamoorthy, J., Wheeler, D., Holla, S., and Grewal, S.I.S. (2021). Spreading and epigenetic inheritance of heterochromatin require a critical density of histone H3 lysine 9 tri-methylation. Proc. Natl. Acad. Sci. USA *118*, e2100699118. https://doi.org/10.1073/pnas.2100699118.

Dobin, A., Davis, C.A., Schlesinger, F., Drenkow, J., Zaleski, C., Jha, S., Batut, P., Chaisson, M., and Gingeras, T.R. (2013). STAR: ultrafast universal RNA-seq aligner. Bioinformatics *29*, 15–21. https://doi.org/10.1093/BIOINFORMATICS/BTS635.

Escobar, T.M., Loyola, A., and Reinberg, D. (2021). Parental nucleosome segregation and the inheritance of cellular identity. Nat. Rev. Genet. *22*, 379–392. https://doi.org/10.1038/S41576-020-00312-W.

Fritsch, L., Robin, P., Mathieu, J.R.R., Souidi, M., Hinaux, H., Rougeulle, C., Harel-Bellan, A., Ameyar-Zazoua, M., and Ait-Si-Ali, S. (2010). A subset of the histone H3 lysine 9 methyltransferases Suv39h1, G9a, GLP, and SETDB1 participate in a multimeric complex. Mol. Cell *37*, 46–56. https://doi.org/10.1016/j.molcel.2009.12.017.

Greiner, D., Bonaldi, T., Eskeland, R., Roemer, E., and Imhof, A. (2005). Identification of a specific inhibitor of the histone methyltransferase SU(VAR)3-9. Nat. Chem. Biol. *1*, 143–145. https://doi.org/10.1038/nchembio721.

Huang, J., Zhang, H., Yao, J., Qin, G., Wang, F., Wang, X., Luo, A., Zheng, Q., Cao, C., and Zhao, J. (2016). BIX-01294 increases pig cloning efficiency by improving epigenetic reprogramming of somatic cell nuclei. Reproduction *151*, 39–49. https://doi.org/10.1530/REP-15-0460.

Huang, Y., Jiang, X., Yu, M., Huang, R., Yao, J., Li, M., Zheng, F., and Yang, X. (2017). Beneficial effects of diazepin-quinazolin-amine derivative (BIX-01294) on preimplantation development and molecular characteristics of cloned mouse embryos. Reprod. Fertil. Dev. *29*, 1260–1269. https://doi.org/10.1071/RD15463.

Iwasa, E., Hamashima, Y., Fujishiro, S., Higuchi, E., Ito, A., Yoshida, M., and Sodeoka, M. (2010). Total synthesis of (+)-chaetocin and its analogues: their histone methyltransferase g9a inhibitory activity. J. Am. Chem. Soc. *132*, 4078–4079. https://doi.org/10.1021/JA101280P.

Jafarpour, F., Ghazvini Zadegan, F., Ostadhosseini, S., Hajian, M., Kiani-Esfahani, A., and Nasr-Esfahani, M.H. (2020). siRNA inhibition and not chemical inhibition of Suv39h1/2 enhances pre-implantation embryonic development of bovine somatic cell nuclear transfer embryos. PLoS One *15*, e0233880. https://doi.org/10.1371/journal.pone.0233880.

Jeong, P.S., Sim, B.W., Park, S.H., Kim, M.J., Kang, H.G., Nanjidsuren, T., Lee, S., Song, B.S., Koo, D.B., and Kim, S.U. (2020). Chaetocin improves pig cloning efficiency by enhancing epigenetic reprogramming and autophagic activity. Int. J. Mol. Sci. *21*, 4836. https://doi.org/10.3390/ijms21144836.

Kishigami, S., Mizutani, E., Ohta, H., Hikichi, T., Thuan, N.V., Wakayama, S., Bui, H.-T., and Wakayama, T. (2006). Significant improvement of mouse cloning technique by treatment with trichostatin A after somatic nuclear transfer. Biochem. Biophys. Res. Commun. *340*, 183–189. https://doi.org/10.1016/j.bbrc.2005.11.164.

Kubicek, S., O'Sullivan, R.J., August, E.M., Hickey, E.R., Zhang, Q., Teodoro, M.L., Rea, S., Mechtler, K., Kowalski, J.A., Homon, C.A., et al. (2007). Reversal of H3K9me2 by a small-molecule inhibitor for the G9a histone methyltransferase. Mol. Cell *25*, 473–481. https://doi.org/10.1016/j.molcel.2007.01.017.

Lehár, J., Krueger, A.S., Avery, W., Heilbut, A.M., Johansen, L.M., Price, E.R., Rickles, R.J., Short, G.F., 3rd, Staunton, J.E., Jin, X., et al. (2009). Synergistic drug combinations tend to improve therapeutically relevant selectivity. Nat. Biotechnol. *27*, 659–666. https://doi.org/10.1038/nbt.1549.

Liao, Y., Smyth, G.K., and Shi, W. (2014). featureCounts: an efficient general purpose program for assigning sequence reads to genomic features. Bioinformatics *30*, 923–930. https://doi.org/10.1093/BIOINFORMATICS/BTT656.

Liu, W., Liu, X., Wang, C., Gao, Y., Gao, R., Kou, X., Zhao, Y., Li, J., Wu, Y., Xiu, W., et al. (2016). Identification of key factors conquering developmental arrest of somatic cell cloned embryos by combining embryo biopsy and single-cell sequencing. Cell Discov. *2*, 16010. https://doi.org/10.1038/celldisc.2016.10.

Liu, Z., Cai, Y., Wang, Y., Nie, Y., Zhang, C., Xu, Y., Zhang, X., Lu, Y., Wang, Z., Poo, M., and Sun, Q. (2018). Cloning of macaque monkeys by somatic cell nuclear transfer. Cell *172*, 881–887.e7. https://doi.org/10.1016/j.cell.2018.01.020.

Love, M.I., Huber, W., and Anders, S. (2014). Moderated estimation of fold change and dispersion for RNA-seq data with DESeq2. Genome Biol. *15*, 550. https://doi.org/10.1186/S13059-014-0550-8.

Matoba, S., and Zhang, Y. (2018). Somatic cell nuclear transfer reprogramming: mechanisms and applications. Cell Stem Cell *23*, 471–485. https://doi.org/10.1016/j.stem.2018.06.018.

Matoba, S., Liu, Y., Lu, F., Iwabuchi, K.A., Shen, L., Inoue, A., and Zhang, Y. (2014). Embryonic development following somatic cell nuclear transfer impeded by persisting histone methylation. Cell *159*, 884–895. https://doi.org/10.1016/j.cell.2014.09.055.

Matoba, S., Wang, H., Jiang, L., Lu, F., Iwabuchi, K.A., Wu, X., Inoue, K., Yang, L., Press, W., Lee, J.T., et al. (2018). Loss of H3K27me3 imprinting in somatic cell nuclear transfer embryos disrupts post-implantation development. Cell Stem Cell *23*, 343–354.e5. https://doi.org/10.1016/j.stem.2018.06.008.

Nishigaya, Y., Takase, S., Sumiya, T., Kikuzato, K., Sato, T., Niwa, H., Sato, S., Nakata, A., Sonoda, T., Hashimoto, N., et al. (2023). Discovery of novel substrate-competitive lysine methyltransferase G9a inhibitors as anticancer agents. J. Med. Chem. *66*, 4059–4085. https://doi.org/10.1021/acs.jmedchem.2c02059.

Padeken, J., Methot, S.P., and Gasser, S.M. (2022). Establishment of H3K9-methylated heterochromatin and its functions in tissue differentiation and maintenance. Nat. Rev. Mol. Cell Biol. *23*, 623–640. https://doi.org/10.1038/s41580-022-00483-w.

Peters, A.H., O'Carroll, D., Scherthan, H., Mechtler, K., Sauer, S., Schöfer, C., Weipoltshammer, K., Pagani, M., Lachner, M., Kohlmaier, A., et al. (2001). Loss of the Suv39h histone methyltransferases impairs mammalian heterochromatin and genome stability. Cell *107*, 323–337.

Pinheiro, I., Margueron, R., Shukeir, N., Eisold, M., Fritzsch, C., Richter, F.M., Mittler, G., Genoud, C., Goyama, S., Kurokawa, M., et al. (2012). Prdm3 and Prdm16 are H3K9me1 methyltransferases required for mammalian heterochromatin integrity. Cell *150*, 948–960. https://doi.org/10.1016/j.cell.2012.06.048.

Rice, J.C., and Allis, C.D. (2001). Histone methylation versus histone acetylation: new insights into epigenetic regulation. Curr. Opin. Cell Biol. *13*, 263–273. https://doi.org/10.1016/S0955-0674(00)00208-8.

Rybouchkin, A., Kato, Y., and Tsunoda, Y. (2006). Role of histone acetylation in reprogramming of somatic nuclei following nuclear transfer. Biol. Reprod. *74*, 1083–1089. https://doi.org/10.1095/biolreprod.105.047456.

Sankar, A., Lerdrup, M., Manaf, A., Johansen, J.V., Gonzalez, J.M., Borup, R., Blanshard, R., Klungland, A., Hansen, K., Andersen, C.Y., et al. (2020). KDM4A regulates the maternal-to-zygotic transition by protecting broad H3K4me3 domains from H3K9me3 invasion in oocytes. Nat. Cell Biol. *22*, 380–388. https://doi.org/10.1038/s41556-020-0494-z.

Shan, C.M., Fang, Y., and Jia, S. (2023). Leaving histone unturned for epigenetic inheritance. FEBS J. *290*, 310–320. https://doi.org/10.1111/FEBS.16260.

Shi, Y., and Whetstine, J.R. (2007). Dynamic regulation of histone lysine methylation by demethylases. Mol. Cell *25*, 1–14. https://doi.org/10.1016/j.molcel.2006.12.010.

Sweis, R.F., Pliushchev, M., Brown, P.J., Guo, J., Li, F., Maag, D., Petros, A.M., Soni, N.B., Tse, C., Vedadi, M., et al. (2014). Discovery and development of potent and selective inhibitors of histone methyltransferase G9a. ACS Med. Chem. Lett. *5*, 205–209. https://doi.org/10.1021/ml400496h.

Tachibana, M., Sugimoto, K., Nozaki, M., Ueda, J., Ohta, T., Ohki, M., Fukuda, M., Takeda, N., Niida, H., Kato, H., and Shinkai, Y. (2002). G9a histone methyltransferase plays a dominant role in euchromatic histone H3 lysine 9 methylation and is essential for early embryogenesis. Genes Dev. *16*, 1779–1791. https://doi.org/10.1101/gad.989402.

Tachibana, M., Ueda, J., Fukuda, M., Takeda, N., Ohta, T., Iwanari, H., Sakihama, T., Kodama, T., Hamakubo, T., and Shinkai, Y. (2005). Histone methyltransferases G9a and GLP form heteromeric

complexes and are both crucial for methylation of euchromatin at H3-K9. Genes Dev. *19*, 815–826. https://doi.org/10.1101/gad.1284005.

Takase, S., Hiroyama, T., Shirai, F., Maemoto, Y., Nakata, A., Arata, M., Matsuoka, S., Sonoda, T., Niwa, H., Sato, S., et al. (2023). A specific G9a inhibitor unveils BGLT3 lncRNA as a universal mediator of chemically induced fetal globin gene expression. Nat. Commun. *14*, 23. https://doi.org/10.1038/s41467-022-35404-0.

Towbin, B.D., González-Aguilera, C., Sack, R., Gaidatzis, D., Kalck, V., Meister, P., Askjaer, P., and Gasser, S.M. (2012). Step-wise methylation of histone H3K9 positions heterochromatin at the nuclear periphery. Cell *150*, 934–947. https://doi.org/10.1016/j.cell.2012.06.051.

Vedadi, M., Barsyte-Lovejoy, D., Liu, F., Rival-Gervier, S., Allali-Hassani, A., Labrie, V., Wigle, T.J., DiMaggio, P.A., Wasney, G.A., Siarheyeva, A., et al. (2011). A chemical probe selectively inhibits G9a and GLP methyltransferase activity in cells. Nat. Chem. Biol. *7*, 566–574. https://doi.org/10.1038/nchembio.599.

Whetstine, J.R., Nottke, A., Lan, F., Huarte, M., Smolikov, S., Chen, Z., Spooner, E., Li, E., Zhang, G., Colaiacovo, M., and Shi, Y. (2006). Reversal of histone lysine trimethylation by the JMJD2 family of histone demethylases. Cell *125*, 467–481. https://doi.org/10.1016/j.cell.2006.03.028.

Zhang, Y.M., Gao, E.E., Wang, Q.Q., Tian, H., and Hou, J. (2018). Effects of histone methyltransferase inhibitor chaetocin on histone H3K9 methylation of cultured ovine somatic cells and development of preimplantation cloned embryos. Reprod. Toxicol. *79*, 124–131. https://doi.org/10.1016/J.REPROTOX.2018.06.006.

Stem Cell Reports, Volume *19*

Supplemental Information

Reduction of H3K9 methylation by G9a inhibitors improves the development of mouse SCNT embryos

Shogo Matoba, Daiki Shikata, Fumiyuki Shirai, Takaki Tatebe, Michiko Hirose, Akiko Nakata, Naomi Watanabe, Ayumi Hasegawa, Akihiro Ito, Minoru Yoshida, and Atsuo Ogura

Supplemental experimental procedures

In vitro fertilization
B6N female and DBA/2 male mice at the age of 9–12 weeks were used to generate IVF control embryos in a BDF1 background. Briefly, spermatozoa were harvested from the epididymis of adult DBA/2 males and incubated in human tubal fluid (HTF) drops for 1 h at 37°C under 5% CO_2 in humidified air. Cumulus–oocyte complexes (COCs) were collected from the oviducts of B6N females that had been superovulated by injection of anti-inhibin serum (gift of Dr. Gen Watanabe at Tokyo University of Agriculture and Technology) followed by 7.5 IU of human chorionic gonadotropin (hCG; Aska Pharmaceutical Co. Ltd.) at 48 h intervals. The COCs were isolated 15–17 h after the hCG injection and incubated in HTF containing 0.04% glutathione (L-glutathione reduced, Sigma Aldrich #G6013) for 1 h before insemination. After preincubation, the activated spermatozoa were introduced into HTF drops containing COCs to initiate insemination. Five to six hours after the initiation of insemination, the fertilized zygotes were washed and transferred to drops of potassium-enriched simplex optimization medium (KSOM).

Donor cell preparation
Primary MEFs were derived from BDF1 male mouse embryos at 13.5 days post coitum. After removal of the head and all organs, minced tissue from the remaining corpus was dissociated in 500 ml of 0.25% trypsin with 1 mM EDTA (Thermo Fisher Scientific #25200056) for 10 min at 37°C. The cell suspension was washed twice with 10% fetal bovine serum (FBS) DMEM and plated in 100-mm dishes, and the plates were cultured at 37°C. Two days later, MEFs were harvested and frozen. Frozen stocks of MEFs were thawed and used for experiments after one passage.
Cumulus cells were collected from adult BDF1 female mice treated with superovulation by injection of 7.5 IU of pregnant mare serum gonadotropin (PMSG; Millipore #367222) and 7.5 IU of hCG (Millipore #230734). COCs were collected from the oviducts 15–17 h after the hCG injection and were treated briefly with HEPES-buffered KSOM containing 300 U/ml bovine testicular hyaluronidase (Calbiochem #385931) to obtain dissociated cumulus cells.
Sertoli cells were collected from the testes of 3–7-day-old BDF1 male mice as described previously (Matoba et al., 2011, 2018). Testicular masses were incubated in phosphate-buffered saline (PBS) containing 0.1 mg/ml collagenase (Thermo Fisher Scientific #17104-019) for 30 min at 37°C and then treated for 5 min with 0.25% trypsin with 1 mM EDTA at room temperature. The dissociated cells were washed four times with PBS containing 3 mg/ml bovine serum albumin (BSA) and then suspended in HEPES-buffered KSOM medium.

Embryo transfer
Two-cell stage SCNT embryos were transferred to the oviducts of pseudopregnant (embryonic day (E) 0.5) ICR females. The pups were recovered by caesarean section on the day of delivery (E19.5) and nursed by lactating ICR females.

Reverse Transcription and Real-Time PCR (related to Figure S1B and S1C)
Two-cell stage embryos were collected at 28 hpi or hpa. Five embryos were pooled as a single sample for each condition. cDNA was directly synthesized with a Cells-to-cDNA kit (Thermo Fisher Scientific, AM1722) with oligo-dT primer. RT-qPCR was then performed using PowerUp SYBR Green Master Mix (#A25742; Thermo Fisher Scientific) with the QuantStudio 7 system

(Thermo Fisher Scientific). The Ct values were normalized to that of external control RNA (Lambda polyA; Takara, #3789). The expression level of each gene was further normalized for IVF control as 1. The primers used for RT-qPCR are as follows; Zscan4d-RT-F1: TCTTTCTGGTTGGCAGCTTT, Zscan4d-RT-R1: GCCTCTGTCAGGACCACTGT, Zscan4a-RT-F1: CCTTTCATCCCACCAGAGAA, Zscan4a-RT-R1: AAGGCTTTTCTGGCATGTGT, Obox3-RT-F1: AGATGAGCTCTGGCAAAGGA, Obox3-RT-R1: TTGGACTGGTCAAGGACCTC, Obox6-RT-F1: TCCTGATTCCTTACCCGTTG, Obox6-RT-R1: CCTGATAATGAGGCGGAGAA, Obox8-RT-F1: CACCCCCAAGAACGTCTAAA, Obox8-RT-R1: GCTAGGGTTGGGTCACAGAA.

Histology (related to Figure S6F)
Placentae collected from E19.5 embryos derived from IVF or SCNT were fixed in 4% paraformaldehyde (PFA) at 4°C overnight and embedded in paraffin blocks. Sections (4 mm in thickness) were subjected to periodic acid Schiff (PAS) staining. Each stained section was scanned on a BZ-9000 microscope (Keyence Japan) and processed using BZ-II Analyzer software (Keyence Japan). At least three placentae for each condition were examined. Representative images of the placenta for each sample were combined into a single panel by adjusting the scale (Figure S6F).

Supplemental Figures

Figure S1

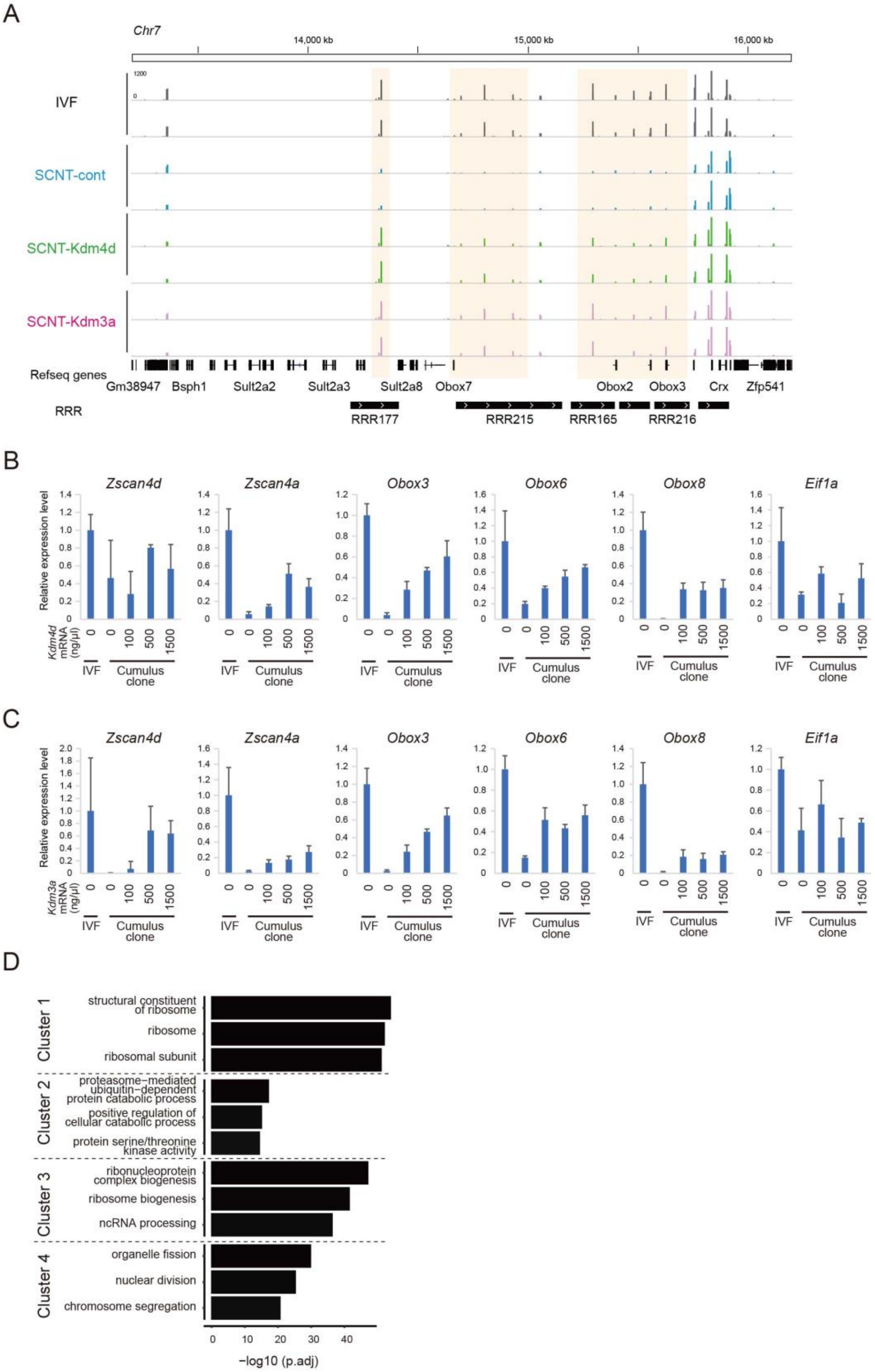

Figure S1. Effect of Kdm3a on the transcriptome of SCNT embryos (related to Figure 2)

(A) A genome browser view of RNA-seq data at the *Obox* cluster on chromosome 7 containing *Kdm3a/4d*-responsive genes (yellow box).

(B) Gene expression levels of ZGA genes in the *Kdm4d* -injected SCNT embryos examined by RT-qPCR. The expression levels were normalized to external control RNA. The value in IVF was set as 1.0.

(C) Gene expression levels of ZGA genes in the *Kdm3a* -injected SCNT embryos examined by RT-qPCR. The expression levels were normalized to external control RNA. The value in IVF was set as 1.0.

(D) Gene ontology analysis of the four clusters classified in Figure 2D.

Figure S2

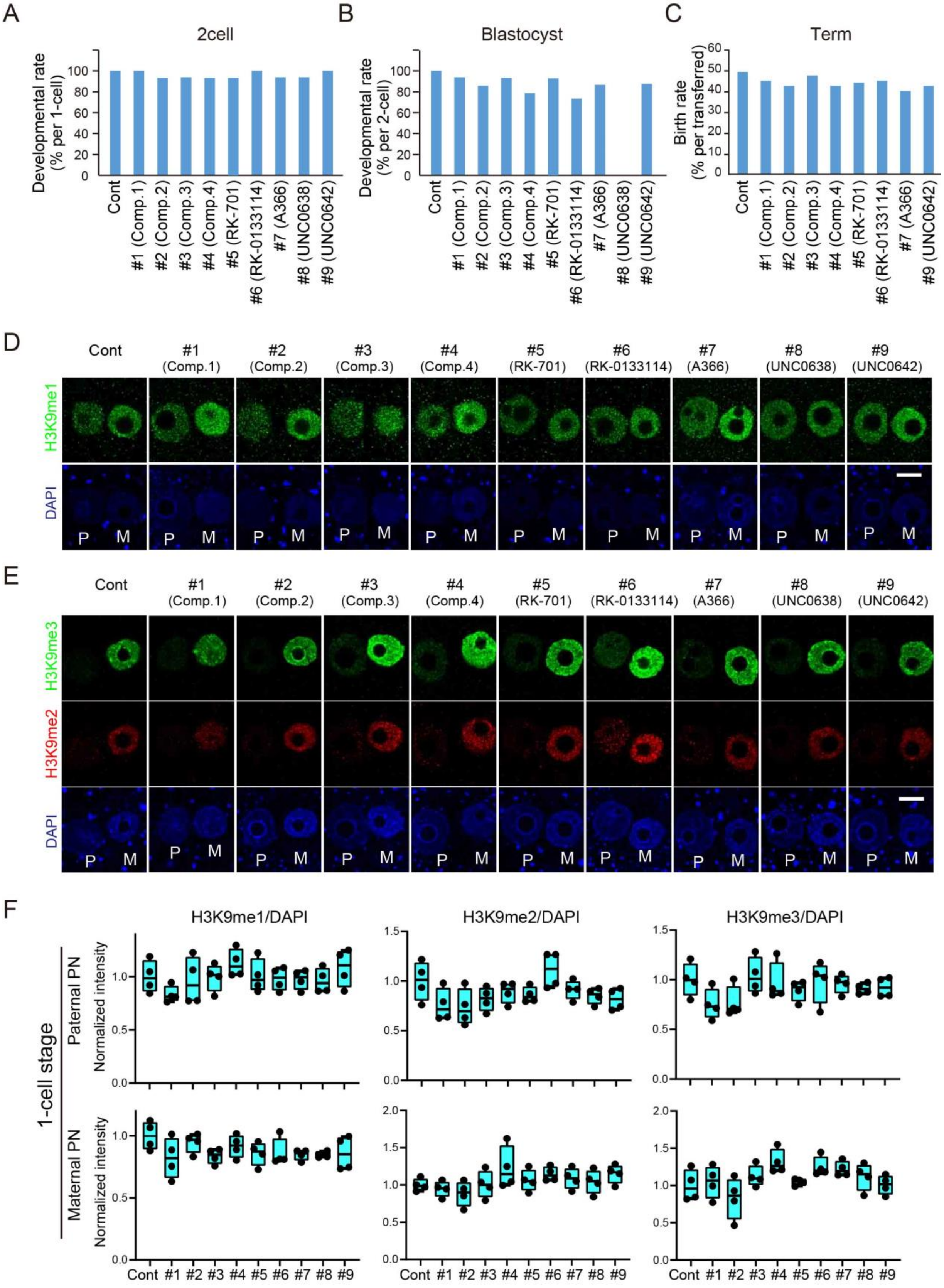
A
2cell
Developmental rate (% per 1-cell)
B
Blastocyst
Developmental rate (% per 2-cell)
C
Term
Birth rate (% per transferred)
Cont
#1 (Comp.1)
#2 (Comp.2)
#3 (Comp.3)
#4 (Comp.4)
#5 (RK-701)
#6 (RK-0133114)
#7 (A366)
#8 (UNC0638)
#9 (UNC0642)
D
H3K9me1
DAPI
P
M
E
H3K9me3
H3K9me2
F
H3K9me1/DAPI
H3K9me2/DAPI
H3K9me3/DAPI
1-cell stage
Paternal PN
Maternal PN
Normalized intensity

Figure S2. Effects of G9a inhibitors on the development and H3K9 methylation levels of IVF embryos (related to Figure 4)

(A, B) Bar graphs showing the developmental rate to the two-cell stage (A), and the blastocyst stage (B). Embryos were treated with the G9a inhibitors indicated at 1 μM concentration from 5 hpi throughout the cultivation period. RK-0133114 is the inactive *R*-enantiomer form of RK-701. All G9ai used in this figure are listed in Table S6.

(C) Bar graphs showing the developmental rate to term. Embryos were treated with the G9a inhibitors indicated at 1 μM concentration from 5 hpi until 24hpi. The embryos were transferred to the oviduct of pseudopregnant females at the 2-cell stage.

(D) Representative images of IVF embryos at the one-cell stage stained with anti-H3K9me1 antibody and DAPI. P, paternal pronucleus. M, maternal pronucleus. Scale bar represents 10 μm.

(E) Representative images of IVF embryos at the one-cell stage stained with anti-H3K9me2 and anti-H3K9me3 antibodies and DAPI. P, paternal pronucleus. M, maternal pronucleus. Scale bar represents 10 μm.

(F) Box plots comparing the relative intensities of H3K9me1, H3K9me2, and H3K9me3 in the IVF one-cell stage embryos. Each signal intensity was normalized to the DAPI signal levels. Four to five embryos were analyzed for each condition.

Figure S3

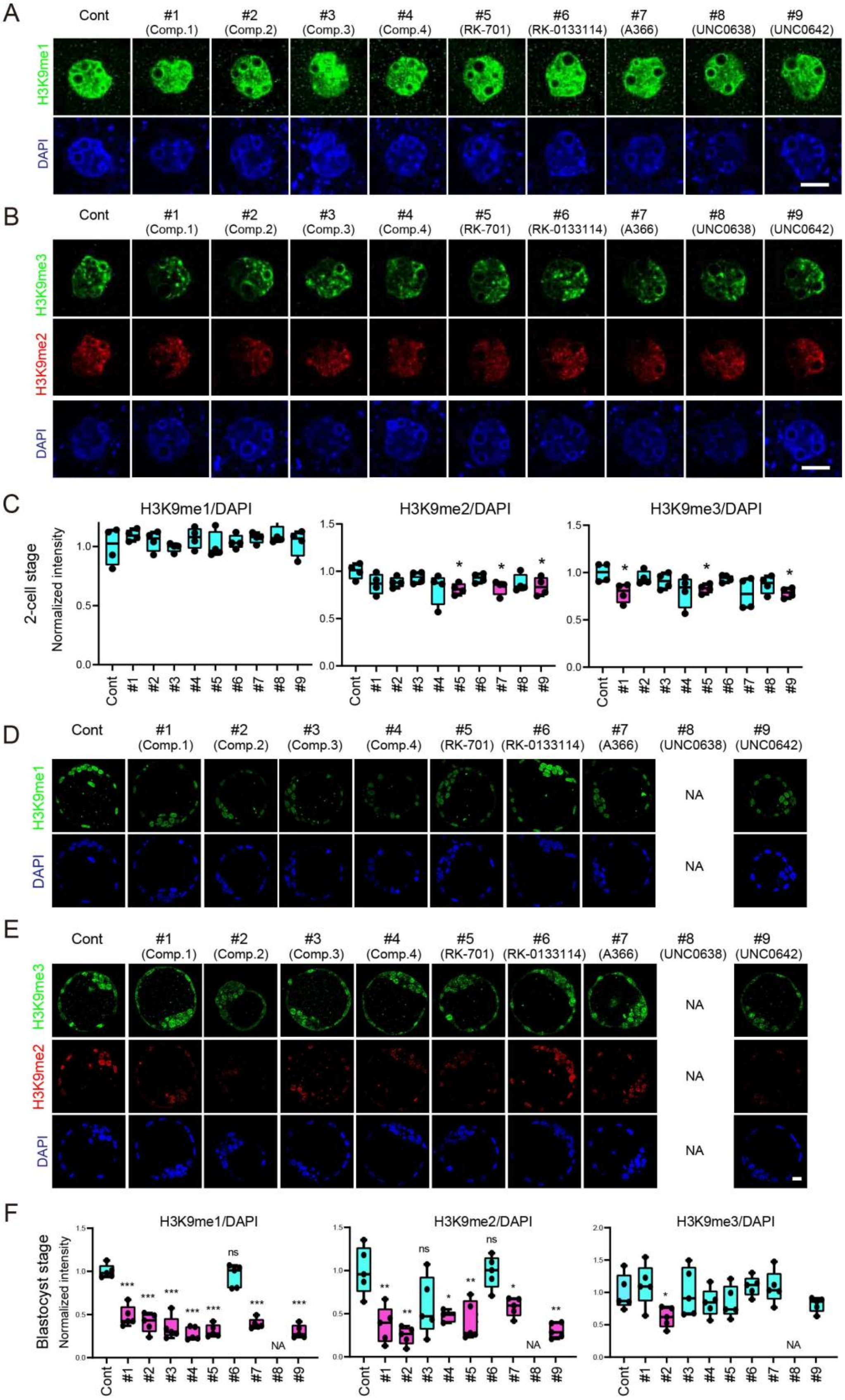
A
Cont
#1 (Comp.1)
#2 (Comp.2)
#3 (Comp.3)
#4 (Comp.4)
#5 (RK-701)
#6 (RK-0133114)
#7 (A366)
#8 (UNC0638)
#9 (UNC0642)
H3K9me1
DAPI
B
H3K9me3
H3K9me2
DAPI
C
H3K9me1/DAPI
H3K9me2/DAPI
H3K9me3/DAPI
2-cell stage
Normalized intensity
D
NA
E
F
Blastocyst stage
ns
NA

Figure S3. Effects of G9a inhibitors on the H3K9 methylation levels of IVF embryos (related to Figure 4)

(A) Representative images of IVF embryos at the two-cell stage stained with anti-H3K9me1 antibody and DAPI. Scale bar represents 10 μm.

(B) Representative images of IVF embryos at the two-cell stage stained with anti-H3K9me2 and anti-H3K9me3 antibodies and DAPI. Scale bar represents 10 μm.

(C) Box plots comparing the relative intensities of H3K9me1, H3K9me2, and H3K9me3 in the IVF two-cell stage embryos. Each signal intensity was normalized to the DAPI signal levels. Four to five embryos were analyzed for each condition. *$P < 0.05$ compared with the control.

(D) Representative images of IVF embryos at the blastocyst stage stained with anti-H3K9me1 antibody and DAPI. Scale bar represents 10 μm.

(E) Representative images of IVF embryos at the blastocyst stage stained with anti-H3K9me2 and anti-H3K9me3 antibodies and DAPI. Scale bar represents 10 μm.

(F) Box plots comparing the relative intensities of H3K9me1, H3K9me2, and H3K9me3 in the IVF blastocysts. Each signal intensity was normalized to the DAPI signal levels. Five embryos were analyzed for each condition. *$P < 0.05$, **$P < 0.01$ ***$P < 0.001$ compared with the control.

Figure S4

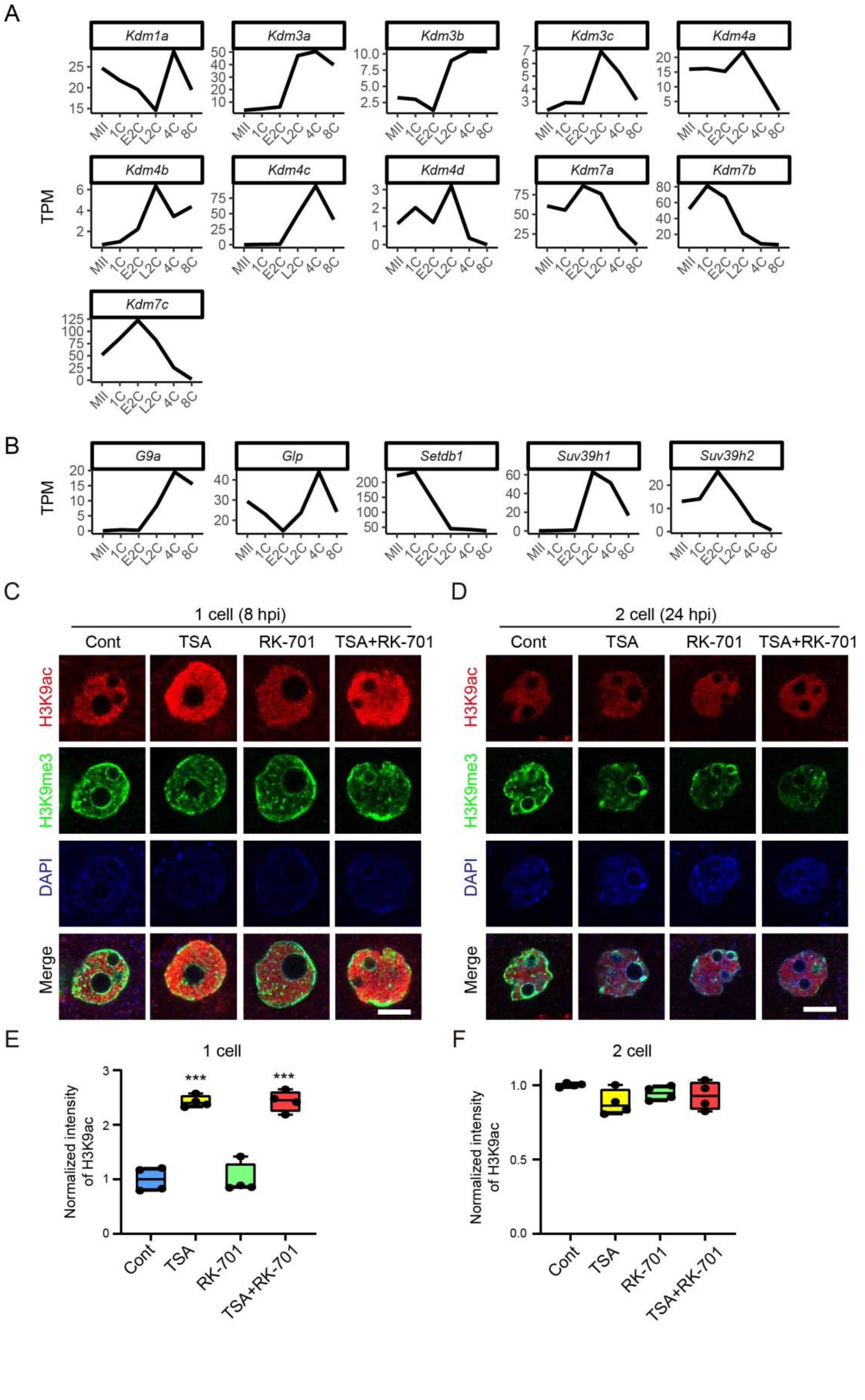
A
Kdm1a
Kdm3a
Kdm3b
Kdm3c
Kdm4a
Kdm4b
Kdm4c
Kdm4d
Kdm7a
Kdm7b
Kdm7c
TPM
MII 1C E2C L2C 4C 8C
B
G9a
Glp
Setdb1
Suv39h1
Suv39h2
TPM
C
1 cell (8 hpi)
Cont TSA RK-701 TSA+RK-701
D
2 cell (24 hpi)
Cont TSA RK-701 TSA+RK-701
H3K9ac
H3K9me3
DAPI
Merge
E
1 cell
***
***
Normalized intensity of H3K9ac
Cont TSA RK-701 TSA+RK-701
F
2 cell
Normalized intensity of H3K9ac
Cont TSA RK-701 TSA+RK-701

Figure S4. Gene expression levels of histone methyltransferases and demethylases for H3K9me2 in the mouse preimplantation embryos and H3K9 acetylation levels in the SCNT embryos (related to Figure 4)

(A) Line plots showing the gene expression levels of histone demethylases with H3K9 demethylation activities in the mouse preimplantation embryos. The gene expression data was obtained from GSE71434 (Zhang et al., 2016).

(B) Line plots showing the gene expression levels of histone methyltransferases for H3K9 deposition in the mouse preimplantation embryos. The gene expression data was obtained from GSE71434 (Zhang et al., 2016).

(C) Representative images of SCNT embryos at the one-cell stage stained with anti-H3K9ac and anti-H3K9me3 antibodies and DAPI. Scale bar represents 10 μm.

(D) Representative images of SCNT embryos at the two-cell stage stained with anti-H3K9ac and anti-H3K9me3 antibodies and DAPI. Scale bar represents 10 μm.

(E) Box plots comparing the normalized intensities of H3K9ac in the SCNT embryos at the one-cell stage. Each signal intensity was normalized to the DAPI signal levels. Four embryos were analyzed for each condition. ***$P < 0.001$ compared with the control.

(F) Box plots comparing the normalized intensities of H3K9ac in the SCNT embryos at the two-cell stage. Each signal intensity was normalized to the DAPI signal levels. Four embryos were analyzed for each condition.

Figure S5

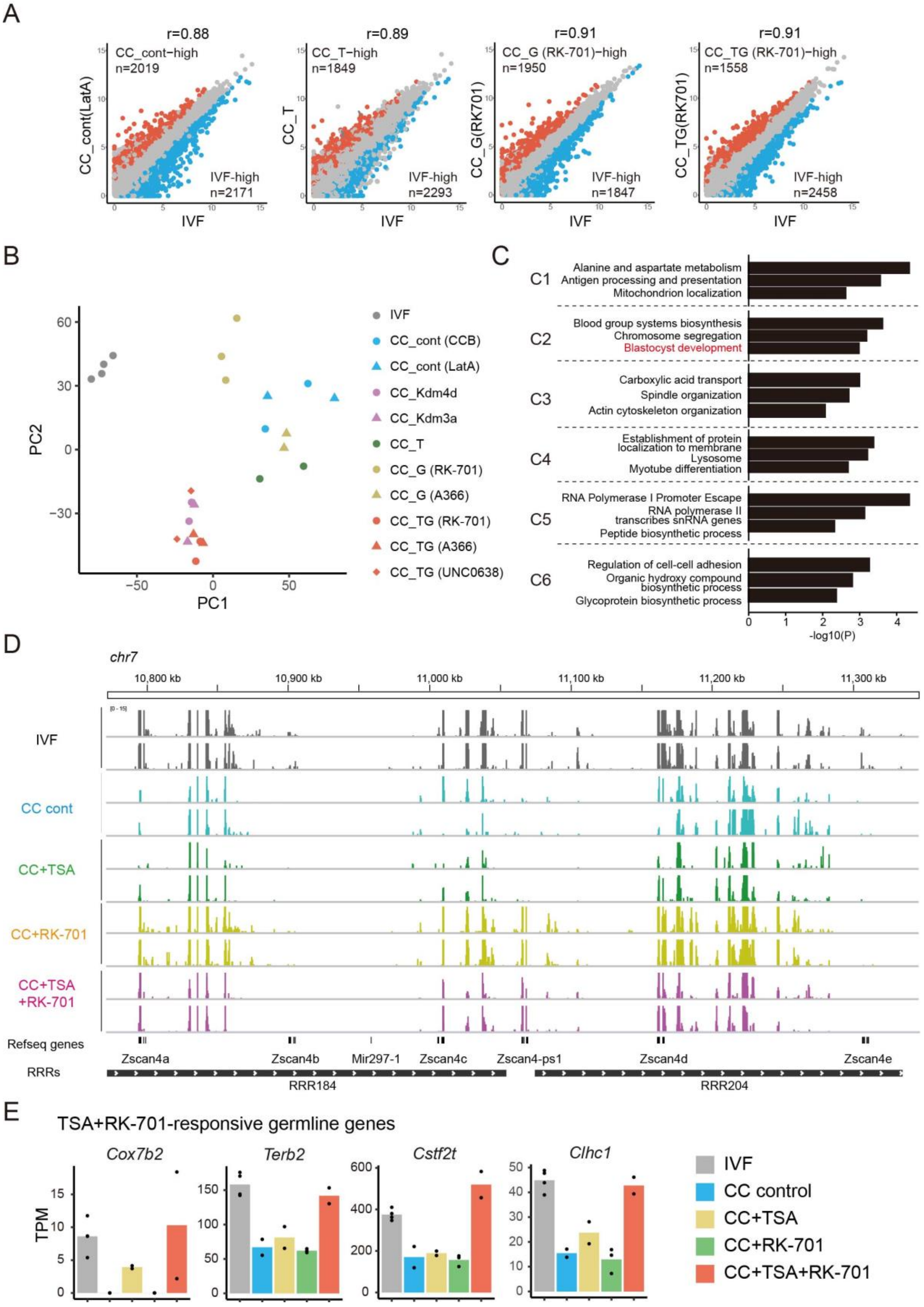

A
r=0.88
CC_cont−high
n=2019
CC_cont(LatA)
IVF-high
n=2171
IVF
r=0.89
CC_T−high
n=1849
CC_T
IVF-high
n=2293
r=0.91
CC_G (RK-701)−high
n=1950
CC_G(RK701)
IVF-high
n=1847
r=0.91
CC_TG (RK-701)−high
n=1558
CC_TG(RK701)
IVF-high
n=2458
B
PC1
PC2
IVF
CC_cont (CCB)
CC_cont (LatA)
CC_Kdm4d
CC_Kdm3a
CC_T
CC_G (RK-701)
CC_G (A366)
CC_TG (RK-701)
CC_TG (A366)
CC_TG (UNC0638)
C
C1
Alanine and aspartate metabolism
Antigen processing and presentation
Mitochondrion localization
C2
Blood group systems biosynthesis
Chromosome segregation
Blastocyst development
C3
Carboxylic acid transport
Spindle organization
Actin cytoskeleton organization
C4
Establishment of protein localization to membrane
Lysosome
Myotube differentiation
C5
RNA Polymerase I Promoter Escape
RNA polymerase II transcribes snRNA genes
Peptide biosynthetic process
C6
Regulation of cell-cell adhesion
Organic hydroxy compound biosynthetic process
Glycoprotein biosynthetic process
-log10(P)
D
chr7
10,800 kb
10,900 kb
11,000 kb
11,100 kb
11,200 kb
11,300 kb
IVF
CC cont
CC+TSA
CC+RK-701
CC+TSA +RK-701
Refseq genes
Zscan4a
Zscan4b
Mir297-1
Zscan4c
Zscan4-ps1
Zscan4d
Zscan4e
RRRs
RRR184
RRR204
E
TSA+RK-701-responsive germline genes
Cox7b2
Terb2
Cstf2t
Clhc1
TPM
IVF
CC control
CC+TSA
CC+RK-701
CC+TSA+RK-701

Figure S5. Effect of G9ai on the transcriptome of SCNT embryos at the two-cell stage (related to Figure 5)

(A) Scatterplots comparing the gene expression levels of IVF and SCNT embryos at the two-cell stage. The genes with significant differences (FC > 2) are colored red or blue.

(B) PCA plot of the transcriptome derived from the two-cell stage embryos. Genes with TPM > 1 on average among all samples were used. Each dot represents a single sample. Note that G9ai and TSA synergistically converted the transcriptome of SCNT embryos and that three different G9ai-treated samples co-treated with TSA (CC_TG (RK-701), CC_TG (A366), and CC_TG (UNC0638)) were closely clustered together with *Kdm3a*- and *Kdm4d*-injected embryos.

(C) Gene ontology analysis of the six clusters (C1: Cluster 1) classified in Figure 5B. Note that the “Blastocyst development” term was enriched in C2.

(D) A genome browser view of RNA-seq data at the *Zscan4* cluster on chromosome 7.

(E) Bar graphs showing the expression levels of TSA+RK-701-responsive germline genes.

Figure S6

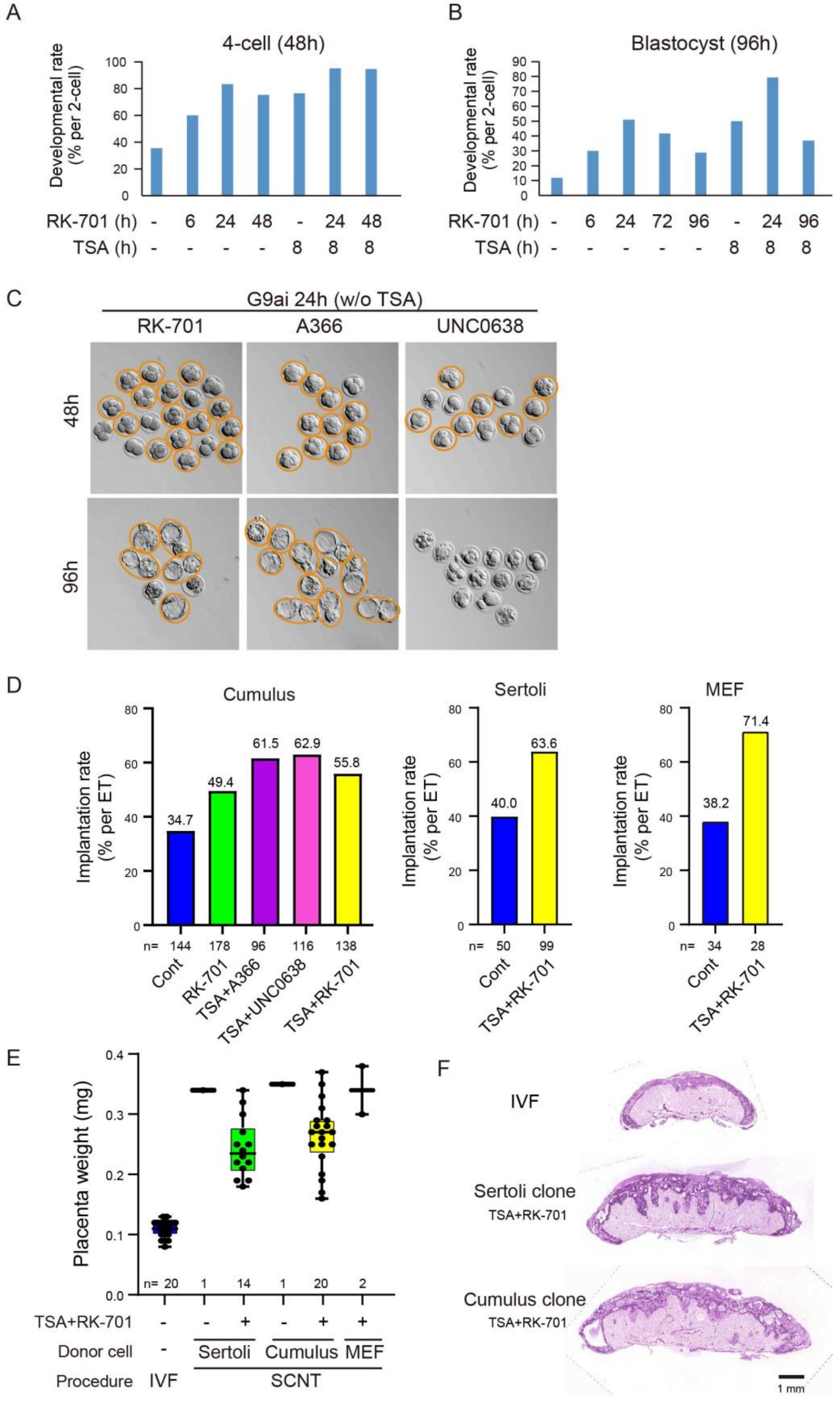
A
4-cell (48h)
Developmental rate (% per 2-cell)
100
80
60
40
20
0
RK-701 (h) - 6 24 48 - 24 48
TSA (h) - - - - 8 8 8
B
Blastocyst (96h)
Developmental rate (% per 2-cell)
90
80
70
60
50
40
30
20
10
0
RK-701 (h) - 6 24 72 96 - 24 96
TSA (h) - - - - - 8 8 8
C
G9ai 24h (w/o TSA)
RK-701
A366
UNC0638
48h
96h
D
Cumulus
Implantation rate (% per ET)
80
60
40
20
0
34.7
49.4
61.5
62.9
55.8
n= 144 178 96 116 138
Cont
RK-701
TSA+A366
TSA+UNC0638
TSA+RK-701
Sertoli
63.6
40.0
n= 50 99
Cont
TSA+RK-701
MEF
71.4
38.2
n= 34 28
Cont
TSA+RK-701
E
Placenta weight (mg)
0.4
0.3
0.2
0.1
0.0
n= 20 1 14 1 20 2
TSA+RK-701 - - + - + +
Donor cell - Sertoli Cumulus MEF
Procedure IVF SCNT
F
IVF
Sertoli clone
TSA+RK-701
Cumulus clone
TSA+RK-701
1 mm

Figure S6. Effects of G9ai on the embryonic development of SCNT embryos (related to Figure 6)

(A, B) Bar graph showing the developmental rate of SCNT embryos derived from cumulus cells at the four-cell stage (A) and the blastocyst stage (B).

(C) Representative images of SCNT embryos derived from cumulus cells at 48 and 96 h of in vitro culture. Normally developing embryos are marked with orange circles. Scale bar, 100 μm.

(D) Bar graphs showing the implantation rate of SCNT embryos examined by the caesarean section on E19.5.

(E) Box plots showing the weight of placentae examined by the caesarean section on E19.5. The whiskers represent the maximum and minimum, and n represents the number of placentae examined.

(F) Representative images of histological sections of a term placenta stained with periodic acid-Schiff (PAS). Microscopic images for each sample were combined into a single panel by adjusting the scale. Note that the PAS-positive spongiotrophoblast layer invaded into the labyrinthine layer in the SCNT placenta even with TSA and RK-701 co-treatment. Scale bar, 1 mm.

Supplemental Tables

Table S1. Gene expression levels in all samples analyzed in this study (related to Figures 2 and 5; see Excel file)

Table S2. Preimplantation development of SCNT embryos (related to Figures 3 and 6)

Donor cell		Name of sample	Actin polymer-ization inhibitor	mRNA injected	G9a inhibitor (1 µM, 24 h)	HDAC inhibitor (25 nM, 8 h)	No. of 1-cell embryos	No. of cleaved embryos (% per 1-cell)	No. of 4-cell embryos (% per cleaved)	No. of 8-cell or morula embryos (% per cleaved)	No. of blastocysts (% per cleaved)
Cell type	Sex										
Cumulus	Female	SCNT_Cont (CCB)	CCB	–	–	–	54	50 (93)	15 (30)	9 (18)	7 (14)
		SCNT_Kdm3a	CCB	*Kdm3a*	–	–	64	60 (94)	56 (93)	48 (80)	42 (70)
		SCNT_Kdm4d	CCB	*Kdm4d*	–	–	60	57 (95)	54 (95)	50 (88)	46 (81)
		CC_Cont (LatA)	LatA	–	–	–	53	49 (92)	17 (35)	9 (18)	8 (16)
		CC_T	LatA	–	–	TSA	58	55 (95)	42 (76)	33 (60)	29 (53)
		CC_G (RK-701)	LatA	–	RK-701	–	63	59 (94)	46 (78)	36 (61)	26 (44)
		CC_TG (RK-701)	LatA	–	RK-701	TSA	78	75 (96)	71 (95)	62 (83)	60 (80)
		CC_TG (A366)	LatA	–	A366	TSA	72	70 (97)	69 (99)	57 (81)	55 (79)
		CC_TG (UNC0638)	LatA	–	UNC0638	TSA	79	74 (94)	72 (97)	67 (91)	63 (85)
Sertoli	Male	SC_Cont	LatA	–	–	–	89	79 (89)	26 (33)	20 (25)	12 (15)
		SC_TG (RK-701)	LatA	–	RK-701	TSA	105	92 (88)	87 (95)	83 (90)	78 (85)
MEF	Male	MEFC_Cont	LatA	–	–	–	55	49 (89)	10 (20)	7 (14)	5 (10)
		MEFC_TG (RK-701)	LatA	–	RK-701	TSA	53	47 (89)	43 (91)	41 (87)	39 (83)

The concentration of injected *Kdm3a* and *Kdm4d* mRNA was 1500 ng/µl. CCB, cytochalasin B. LatA, latrunculin A. HDAC, histone deacetylase. TSA, trichostatin A.

Table S3. Postimplantation development of IVF embryos treated with G9ai (related to Figure 4)

Name of sample	G9a inhibitor (1 μM, 24 h)	No. of 2-cell embryos transferred	No. of recipient females	No. of implanted (% per ET)	No. of pups at birth (% per ET)	Body weight at birth (g ± SD)	Placenta weight at birth (g ± SD)
Control	–	65	4	41 (63.1)	32 (49.2)	1.52 ± 0.13	0.1 ± 0.02
#1	Compound 1	40	2	23 (57.5)	18 (45.0)	1.47 ± 0.12	0.11 ± 0.02
#2	Compound 2	40	2	25 (62.5)	17 (42.5)	1.54 ± 0.12	0.1 ± 0.01
#3	Compound 3	40	2	23 (57.5)	19 (47.5)	1.5 ± 0.11	0.1 ± 0.01
#4	Compound 4	40	2	24 (60.0)	17 (42.5)	1.54 ± 0.11	0.1 ± 0.01
#5	RK-701	50	3	31 (62.0)	22 (44.0)	1.5 ± 0.1	0.11 ± 0.01
#6	RK-0133114	40	2	25 (62.5)	18 (45.0)	1.48 ± 0.13	0.1 ± 0.01
#7	A366	40	2	26 (65.0)	16 (40.0)	1.5 ± 0.1	0.11 ± 0.01
#9	UNC0642	40	2	24 (60.0)	17 (42.5)	1.51 ± 0.08	0.11 ± 0.01

All embryos were generated by IVF using BDF1 females and males. ET, embryo transfer.

Table S4. List of 879 RRGs (related to Figure 5; see Excel file)

Table S5. Postimplantation development of SCNT embryos (related to Figure 6)

Donor cell: Cell type	Donor cell: Sex	Name of sample	G9a inhibitor (1 μM, 24 h)	HDAC inhibitor (25 nM, 8 h)	No. of 2-cell embryos transferred	No. of implanted (% per ET)	No. of pups at birth (% per ET)	No. of pups survived to adults (% per birth)	Body weight at birth (g ± SD)	Placenta weight at birth (g ± SD)
Cumulus	Female	CC_Cont	–	–	144	50 (34.7)	1 (0.7)	1 (100)	1.65	0.35
		CC_RK-701	RK-701	–	178	88 (49.4)	7 (3.9)	6 (85.7)	1.57 ± 0.13	0.29 ± 0.06
		CC_TSA+A366	A366	TSA	96	59 (61.5)	11 (11.5)	10 (90.9)	1.42 ± 0.14	0.27 ± 0.05
		CC_TSA+UNC0638	UNC0638	TSA	116	73 (62.9)	14 (12.1)	12 (85.7)	1.42 ± 0.11	0.27 ± 0.04
		CC_TSA+RK-701	RK-701	TSA	138	77 (55.8)	20 (14.5)	18 (90)	1.48 ± 0.17	0.26 ± 0.06
Sertoli	Male	SC_Cont	–	–	50	20 (40.0)	1 (1.7)	1 (100)	0.34	0.34
		SC_TSA+RK-701	RK-701	TSA	99	63 (63.6)	14 (14.1)	14 (100)	1.43 ± 0.10	0.24 ± 0.05
MEF	Male	MEFC_Cont	–	–	34	13 (38.2)	0 (0.0)	N/A	N/A	N/A
		MEFC_TSA+RK-701	RK-701	TSA	28	20 (71.4)	2 (7.1)	1 (50.0)	1.69 ± 0.06	0.34 ± 0.06

The concentration of injected *Kdm3a* and *Kdm4d* mRNA was 1500 ng/μl. HDAC, histone deacetylase. TSA, trichostatin A. ET, embryo transfer. N/A, not applicable.

Table S6. List of G9a inhibitors used in this study

Name of inhibitor	IC50 to G9a (nM)	Company	Catalog #	Notes	Reference/Patent #
RK-701	23–27	-	NA		(Nishigaya et al., 2023)<br>(Takase et al., 2023)
RK-0133114	3,700	-	NA	*R*-enantiomer of RK-701	(Nishigaya et al., 2023)<br>(Takase et al., 2023)
Compound 1	21	-	#13-1 in patent		#WO2021106988A1
Compound 2	2.0	-	#6-89 in patent		#WO2021106988A1
Compound 3	24	-	#6-32 in patent		#WO2021106988A1
Compound 4	2.9	-	#1-67 in patent		#WO2021106988A1
A366	3.3	Merck	#SML1410		(Sweis et al., 2014)
UNC0638	< 15	Merck	#U4885		(Vedadi et al., 2011)
UNC0642	< 2.5	Merck	#SML1037		(Liu et al., 2013)

Supplemental References

Liu, F., Barsyte-Lovejoy, D., Li, F., Xiong, Y., Korboukh, V., Huang, X.P., Allali-Hassani, A., Janzen, W.P., Roth, B.L., Frye, S. V., et al. (2013). Discovery of an in vivo chemical probe of the lysine methyltransferases G9a and GLP. J. Med. Chem. *56*, 8931–8942. 10.1021/JM401480R.

Matoba, S., Inoue, K., Kohda, T., Sugimoto, M., Mizutani, E., Ogonuki, N., Nakamura, T., Abe, K., Nakano, T., Ishino, F., et al. (2011). RNAi-mediated knockdown of Xist can rescue the impaired postimplantation development of cloned mouse embryos. Proc. Natl. Acad. Sci. U. S. A. *108*, 20621–20626. 10.1073/pnas.1112664108.

Matoba, S., Wang, H., Jiang, L., Lu, F., Iwabuchi, K.A., Wu, X., Inoue, K., Yang, L., Press, W., Lee, J.T., et al. (2018). Loss of H3K27me3 imprinting in somatic cell nuclear transfer embryos disrupts post-implantation development. Cell Stem Cell *23*, 343–354. 10.1016/j.stem.2018.06.008.

Nishigaya, Y., Takase, S., Sumiya, T., Kikuzato, K., Sato, T., Niwa, H., Sato, S., Nakata, A., Sonoda, T., Hashimoto, N., et al. (2023). Discovery of novel substrate-competitive lysine methyltransferase G9a inhibitors as anticancer agents. J. Med. Chem. *66*, 4059–4085. 10.1021/acs.jmedchem.2c02059.

Sweis, R.F., Pliushchev, M., Brown, P.J., Guo, J., Li, F., Maag, D., Petros, A.M., Soni, N.B., Tse, C., Vedadi, M., et al. (2014). Discovery and development of potent and selective inhibitors of histone methyltransferase G9a. ACS Med. Chem. Lett. *5*, 205–209. 10.1021/ml400496h.

Takase, S., Hiroyama, T., Shirai, F., Maemoto, Y., Nakata, A., Arata, M., Matsuoka, S., Sonoda, T., Niwa, H., Sato, S., et al. (2023). A specific G9a inhibitor unveils BGLT3 lncRNA as a universal mediator of chemically induced fetal globin gene expression. Nat. Commun. *14*, 23. 10.1038/s41467-022-35404-0.

Vedadi, M., Barsyte-Lovejoy, D., Liu, F., Rival-Gervier, S., Allali-Hassani, A., Labrie, V., Wigle, T.J., DiMaggio, P.A., Wasney, G.A., Siarheyeva, A., et al. (2011). A chemical probe selectively inhibits G9a and GLP methyltransferase activity in cells. Nat. Chem. Biol. *7*, 566–574. 10.1038/nchembio.599.

Zhang, B., Zheng, H., Huang, B., Li, W., Xiang, Y., Peng, X., Ming, J., Wu, X., Zhang, Y., Xu, Q., et al. (2016). Allelic reprogramming of the histone modification H3K4me3 in early mammalian development. Nature *537*, 553–557. 10.1038/nature19361.
